# Birdsong “Transcriptomics”: Neurochemical Specializations of the Oscine Song System

**DOI:** 10.1371/journal.pone.0003440

**Published:** 2008-10-20

**Authors:** Peter V. Lovell, David F. Clayton, Kirstin L. Replogle, Claudio V. Mello

**Affiliations:** 1 Neurological Sciences Institute, Oregon Health and Science University, Beaverton, Oregon, United States of America; 2 Cell & Developmental Biology, University of Illinois, Urbana, Illinois, United States of America; The Rockefeller University, United States of America

## Abstract

**Background:**

Vocal learning is a rare and complex behavioral trait that serves as a basis for the acquisition of human spoken language. In songbirds, vocal learning and production depend on a set of specialized brain nuclei known as the song system.

**Methodology/Principal Findings:**

Using high-throughput functional genomics we have identified ∼200 novel molecular markers of adult zebra finch HVC, a key node of the song system. These markers clearly differentiate HVC from the general pallial region to which HVC belongs, and thus represent molecular specializations of this song nucleus. Bioinformatics analysis reveals that several major neuronal cell functions and specific biochemical pathways are the targets of transcriptional regulation in HVC, including: 1) cell-cell and cell-substrate interactions (e.g., cadherin/catenin-mediated adherens junctions, collagen-mediated focal adhesions, and semaphorin-neuropilin/plexin axon guidance pathways); 2) cell excitability (e.g., potassium channel subfamilies, cholinergic and serotonergic receptors, neuropeptides and neuropeptide receptors); 3) signal transduction (e.g., calcium regulatory proteins, regulators of G-protein-related signaling); 4) cell proliferation/death, migration and differentiation (e.g., TGF-beta/BMP and p53 pathways); and 5) regulation of gene expression (candidate retinoid and steroid targets, modulators of chromatin/nucleolar organization). The overall direction of regulation suggest that processes related to cell stability are enhanced, whereas proliferation, growth and plasticity are largely suppressed in adult HVC, consistent with the observation that song in this songbird species is mostly stable in adulthood.

**Conclusions/Significance:**

Our study represents one of the most comprehensive molecular genetic characterizations of a brain nucleus involved in a complex learned behavior in a vertebrate. The data indicate numerous targets for pharmacological and genetic manipulations of the song system, and provide novel insights into mechanisms that might play a role in the regulation of song behavior and/or vocal learning.

## Introduction

The emergence of high-throughput functional genomics has made it possible to identify novel relationships between genes, brain, and behavior. Microarray platforms, in particular, have given enormous momentum to the study of brain gene regulation in the contexts of sensory/motor processing, learning, the formation of memories, aging, and the onset of diseases [1]–[6]. In a more naturalistic context, genomics approaches are also being brought to bear on the genetics of sociality and foraging behavior in honey bees [7], [8] and the emergence of adaptive phenotypes and life history traits in African cichlids [9], [10]. Here we apply a similar approach to songbirds, and ask what are the neurochemical specializations of the discrete neural circuitry necessary for the acquisition and production of learned song.

Vocal learning is a rare trait, expressed in just three orders of birds (e.g. hummingbirds, parrots, songbirds), cetaceans, and humans, where it serves as a basis for the acquisition of spoken language. In vocal learning birds, the memorization and production of song share many important parallels with the process of speech acquisition in humans and depends on a set of telencephalic nuclei referred to collectively as the song control system [11]–[13]. Because these song nuclei are thought to be absent in non-learners [14] (e.g. chicken, pigeon), the transcriptional profile of these nuclei in learners may provide valuable insights into the intrinsic physiological properties of the song system, and also help to identify neurochemical specializations that may be important for vocal learning and/or the production of learned song. In addition, the identification of genes that are differentially regulated between song nuclei and their respective pallial and striatal regions in the avian brain may shed new light on the evolutionary and ontogenetic origins of the song system, and in light of a growing awareness of the relatedness of the avian and mammalian telencephalic regions, to studies in mammals [15], [16].

**Figure 1:**
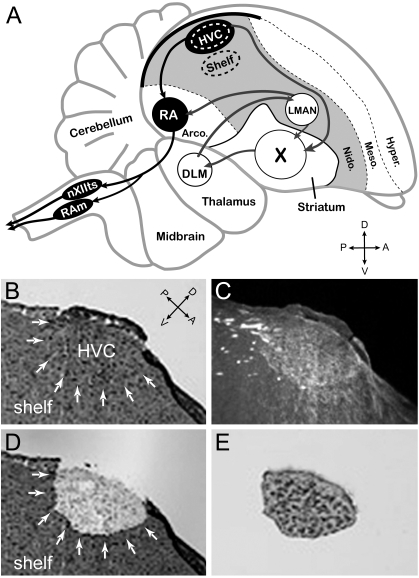
(A) Schematic of a male zebra finch brain showing the approximate locations of HVC and underlying auditory shelf, as well as other major nuclei of the song system and major pallial and subpallial brain divisions. HVC provides major input into: a direct motor pathway (black nuclei), and an anterior forebrain pathway (white nuclei). Laser capture microdissection (LCM) was used to conservatively sample HVC and the underlying auditory shelf (LCM sites denoted by dotted ovals). (B and C) Under brightfield (B, white arrows) HVC could be identified by a characteristic bump on the surface of the brain and the presence of large cells and cell clusters; under dark-field (C) mylenated fibers are seen close to the ventral boundary of HVC. LCM dissections were confirmed by examination of the section after LCM (D), as well as of tissue adhered to the capture cap (E). Abbreviations: A, anterior; P, Posterior; D, Dorsal; V, Ventral; DLM, medial dorsolateral thalamic nucleus; LMAN, lateral magnocellular nucleus of the anterior nidopallium; nXIIts, tracheo-syringeal portion of the hypoglossal nerve nucleus; RA, robust nucleus of the arcopallium; RAm, nucleus retroambigualis; X, striatal area X; *Arco.*, *arcopallium*; *Nido.*, *nidopallum*; *Meso.*, *mesopallium*; *Hyper.*, *hyperpallium*.

In songbirds, the caudo-dorsal portion of the nidopallium (a part of the avian pallium that is thought to share a common origin with mammalian cortical regions) encompasses both HVC, a specialized nucleus of the song system that is unique to songbirds, and the Shelf, a component of the central auditory system that is present in all birds (Fig. 1A) [15]. Our basic hypothesis is that HVC constitutes a differentiated nucleus within the caudo-dorsal nidopallium that is specialized for vocal-motor control and vocal learning, while the Shelf is part of a more primordial avian brain circuitry involved in auditory processing.

HVC provides major inputs to both the direct pathway for vocal-motor control and the anterior forebrain pathway (Fig. 1A), is exquisitely sensitive to sex steroids and sexually dimorphic in many species, and is a major site of neuronal replacement, undergoing marked seasonal fluctuations in size and neuronal composition in seasonal breeders [17]–[19] (see also reviews in [13]). During vocalizations, HVC exhibits characteristic sparse firing, which suggests it is a key structure for motor encoding of learned song, and arguably analogous to pre-motor vocal areas in humans. In contrast, the Shelf region is more intricately involved in auditory processing than vocal-motor control as it receives major inputs from primary auditory areas and originates descending auditory projections [20]. The shelf can also be clearly differentiated from HVC through the differential expression of molecular markers, including a lack of expression of androgen receptors (indicating differential sensitivity to androgen) and the differential induction of activity-dependent genes like *zenk* and *arc* in the contexts of singing (high expression in HVC; [21]) and hearing (high expression in the shelf but not HVC; [22]). While the expression of these and other genes clearly distinguish HVC and Shelf, most currently characterized genes do not differentiate molecularly the Shelf from the rest of the nidopallium, indicating that the properties of the Shelf resemble closely those of this general pallial subdivision. Based on these properties, we predicted that there should be a collection of differentially expressed genes between HVC and Shelf and that the identification of these genes would provide important clues as to molecular and biochemical pathways representing specializations of HVC and possibly involved in different aspects of HVC function.

To identify markers of HVC we used microarray screening of laser capture microdissected samples from HVC and the adjacent Shelf region combined with bioinformatics for a comprehensive analysis of molecular genetic specializations of HVC. Our effort resulted in the identification of over 250 differentially expressed genes that are likely to be related to the unique properties of this song nucleus. Moreover, our bioinformatics analysis provide new perspectives on the roles that individual genes and genetic pathways might play in various aspects of cellular morphogenesis, neurogenesis, steroidogenesis, cellular excitability, neurotransmission, cell survival, steroid and retinoid sensitivity, and gene regulation in HVC.

## Results

### Identification of HVC markers

Despite major progress towards understanding the anatomical and electrophysiological properties of song nucleus HVC, the nature of the molecular mechanisms and biochemical/genetic programs underlying these properties has remained largely unknown. To address this gap, we searched for differentially expressed genes in HVC versus the adjacent Shelf, an auditory area that is also part of the dorso-caudal nidopallium but not part of the song system (Fig. 1A). Throughout the text we use the term “marker” in reference to genes that are differentially expressed between HVC and Shelf, regardless of direction, since genes that have either higher or lower expression in HVC versus Shelf may represent important targets of regulation. Notably, while markers constitute molecular specializations within the context of the HVC/Shelf comparison, this does not preclude their expression and/or enrichment in other brain areas.

To accurately and reproducibly obtain HVC and Shelf samples we used laser capture microscopy of frozen brain sections (Fig. 1B–E). We analyzed 6 adult male zebra finches as independent biological replicates, and used glass microarrays containing an estimated 17,214 unique cDNAs expressed in the zebra finch brain (i.e., ESTIMA:Songbird collection) to identify differentially expressed genes. Notably, we used confirmed non-singing unstimulated zebra finches to minimize the overall expression of genes related to auditory- and/or singing-behavior, and enrich for differentially-expressed genes that might correspond to bona fide, activity-independent markers of HVC. Our microarray hybridizations used a universal design and were conducted as part of Community Collaboration #17 (see Table 7; [23]) under the Songbird Neurogenomics Initiative.

Using conservative ANOVA and protected post-hoc t-tests (Genespring) we identified 390 spots with significant differential expression (FDR<0.05) in HVC as compared to Shelf (Table 1). Since only about 37% of the ESTs for these clones had annotations in ESTIMA at the time of our initial analysis, we aligned each to the chicken genome and performed additional GenBank BlastN searches, resulting in an overall ∼84% annotation rate (see Materials and Methods; clones with tentative identification are underlined in tables and text). Our primary candidate list contained 130 up- and 149 down-regulated non-redundant and 51 redundant clones (Table S1; additional support for gene functions is presented in References S1). An additional 60 clones lacked annotations in ESTIMA, did not share similarity with known genes (NCBI-BLASTN), and failed to align against the chicken genome (Blat, USCS). Providing statistical validation, 93.3% of spots identified as differential in Genespring were also significant according to a separate ANOVA performed using ARDAS at FDR<0.01 (see Materials and Methods for details). A secondary list (Table S2) consisting of markers that were differential in GeneSpring at FDR<0.1 or in 5/6 out of 6 brains and functionally related to genes on our primary list was also generated to supplement the bioinformatics analysis.

**Table 1:** Microarray Results and Candidate HVC Marker Validations.

Differentially Expressed cDNAs (FDR = 0.05)	+	−	ND	Total
Redundant (based on 5′ EST reads)	17	30	NA	47
Non-redundant and unannotated (Unknowns)	31	31	NA	62
Non-redundant and Annotated (Candidate Markers)	130	151	NA	281
*Totals*	*178*	*212*	*-*	*390*
***Candidates Previously Identified as HVC Markers *** †				
Present in Primary Gene list *	6	5	NA	11
Present in Secondary Gene lists **	4	6	3	13
*Subtotals*	*10*	*11*	*3*	*24*
***Candidates Confirmed by In Situ Hybridization*** Δ				
Primary Gene list	21	4	NA	25
Secondary Gene list	1	1	3	5
*Subtotals*	*22*	*5*	*3*	*30*
***Total Confirmed HVC Markers***	***32***	***16***	***3***	***54***

ND = Not Differential.

NA = Not Applicable.

† Previously identified Markers presented in Table S3.

* Primary List (FDR<0.05) presented in Table S1.

** Secondary Lists presented in Tables S2 and S5.

Δ Two genes gave no in situ signal (see text for details).

**Figure 2:**
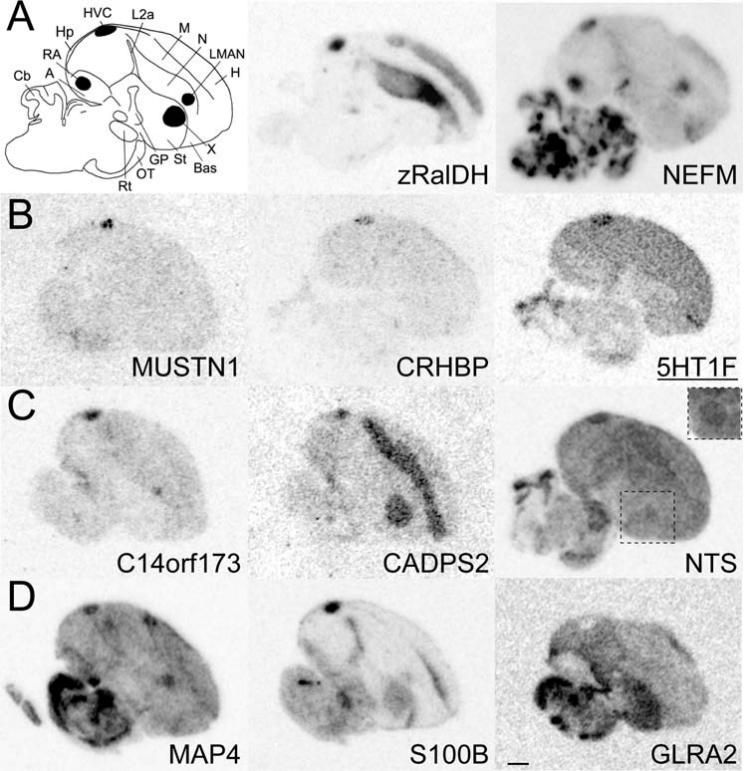
(A–D) Representative *in situ* hybridization autoradiograms of parasagittal sections of adult male zebra finches at the level of HVC (∼1.4 to 2.4 mm from the midline). (A, left) Schematic depicting the major telencephalic song nuclei (in black), thalamorecipient areas (i.e. L2a, Bas), and major brain subdivisions. (A, center and right) Expression of known HVC markers. The other panels (B–D) depict representative autoradiograms of HVC markers that are exclusive to HVC (B), enriched in HVC and other song nuclei, such as LMAN (C, left), or area X (C, center and right; inset shows area X from a more medial section), enriched in 3/4 major song nuclei (D, left and middle), or are negative markers (D, right). Notice that some markers are also enriched in distinct pallial and striatal areas, and/or primary sensory areas. Scalebar: 1 mm. Gene abbreviations in Table S1. Anatomical abbreviations: A, Arcopallium; Bas, nucleus basorostralis; Cb, Cerebellum; GP, globus pallidus; Hp; Hippocampus; H, Hyperpallium; L2a, subfield L2a of field L; LMAN, lateral magnocellular nucleus of the anterior nidopallium; M, Mesopallium; N, Nidopallium; RA, robust nucleus of the arcopallium; Ot, optic tract; Rt, nucleus rotundus; St; striatum.

Providing further validation for our screening, many known HVC markers were present on our primary (n = 11) and secondary (n = 13) lists (presented in Table S3). Among these, retinaldehyde dehydrogenase (*zRalDH*; Fig. 2A, middle; [24]), Calmodulin-binding transcription activator 1 [25], Neurofilament triplet light and medium ([26]; see Fig. 2A, right), Phantom2 [27], insulin-like growth factor 2 [28], voltage-gated potassium channel subfamily C3 (Velho and Mello, unpublished observation), a metabotropic glutamate receptor (GRM8; [25]), the gene *deleted in bladder cancer* (DBC1, a.k.a. brinp1; [29]), and Parvalbumin [30] were all confirmed to be enriched in HVC, whereas P450 aromatase [31], [32], several glutamate receptors subunits (GRIA2, GRIA3, GRIA4, GRIK2, GRM1, and GRM5; [33]), N-chimaerin [34], alpha-synuclein [35], Reelin isoform A [25] and hydroxysteroid (17-beta) dehydrogenase 11/13 ([29]) were all confirmed to have lower expression in HVC, and several glutamate receptor subunits were confirmed as not differential (GRM3, GRIK3, GRIN1; Table S3).

To further validate our screening, we performed *in situ* hybridizations for a subset of candidate markers from our primary list (21 up- and 4 down-regulated), addressing a broad microarray fold-enrichment range (−0.5- to 11.0-fold). We confirmed differential expression between HVC and Shelf for 23 clones (Table S4; examples presented in Fig. 2) and obtained no signal for 2 clones. When combined with the 11 previously known markers of HVC from our primary list, we have confirmed differential expression for 34/34 primary list genes that gave signal, indicating that the vast majority of candidates on this list are bona fide HVC markers. We note that although all these markers were differential between HVC and Shelf, none showed differential expression between Shelf and the caudal nidopallium adjacent to the Shelf.

To determine whether some of our HVC markers might be regulated by singing behavior or revealed by comparisons with different tissues, we cross-referenced our primary gene list against lists from two separate and unrelated microarray studies of HVC. We first compared our list to that derived from a comparison between HVC and the whole brain ([25]; SI Table 5). After removing 412 (52.7%) genes from their list because they lacked unambiguous annotations or were duplicate cDNAs, we determined that ∼31 (23 up and 8 down) of our markers could be categorized as shared between the two studies. This relatively small overlap (∼8%) likely reflects obvious differences in the set of cDNAs spotted on the array platforms as well as the effect of performing comparisons between different tissues (i.e. HVC vs shelf; HVC vs whole brain). Nevertheless, our confirmatory *in situ* analyses indicate that the vast majority of the genes on our list are likely bona fide markers of HVC, representing molecular specializations that clearly differentiate HVC from the adjacent nidopallium. We also compared our list to a list of ∼20 genes shown to be regulated in HVC by singing behavior [36]. We found no shared genes between these lists. Although this does not rule out the possibility that a subset of genes from our primary list may also be regulated by singing, it is consistent with our use of non-singing unstimulated males. Thus, our study has revealed more than 200 annotated and unique genes that have not been previously detected in the context of the oscine song system.

### Expression analysis of HVC markers

Our in situ expression analysis revealed that many HVC markers are also expressed in other song nuclei and brain subdivisions in various combinations (Fig. 2 and Table S4). For example, while nearly 50% of the markers examined (8/21) showed exclusive enrichment in HVC (Fig. 2B), several were also enriched in other song nuclei, including LMAN (Fig. 2C and 2D, left), area X (Fig. 2C, middle and right, see inset), LMAN and area X (e.g. MAPK11, not shown), or all four telencephalic song nuclei (Fig. 2D, middle). Interestingly, some genes (e.g., CADPS2, NTS and S100B) also showed enrichment in distinct pallial and/or primary sensory areas like auditory field L2a and somatosensory nucleus basorostralis; negative markers were confirmed absent in HVC, although expressed in Shelf and other areas (Fig. 2D, right).

**Figure 3:**
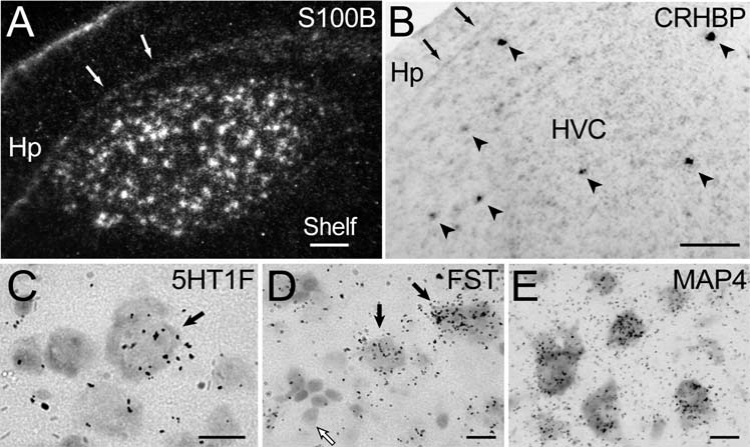
(A and B) Darkfield (A) and brightfield (B) views of nissl stained emulsion autoradiography depicting the expression of S100B (white signal) and CRHBP (black signal and arrowheads) in HVC, respectively; arrows delineate the ventricle. (D) High magnification bright-field views of emulsion autoradiography depicting examples of high expression (black grains) over the majority of large HVC cells (C; Arrows), a portion of these cells (D), or virtually all large and small cells (E) in sections hybridized with antisense probes directed against follistatin (FST), a serotonin receptor (5HT1F), and a microtubule associated protein 4 (MAP4), respectively. White arrow in C indicates an example of a non-labeled small cell. Scalebars: 100 µm in A and B; and 10 µm in C–E. See Fig. 2 legend for anatomical abbreviations.

Emulsion analysis further revealed that HVC markers have distinct cellular distributions. For example, S100B is expressed in a subset of cells that are uniformly distributed throughout HVC (Fig. 2D, middle and Fig. 3A) and in the ventricular zone (Fig. 3A, arrows). In contrast, CRHBP (Fig. 2B middle) is expressed in a very distinct and sparse subset of cells (Fig. 3B), whereas follistatin (FST) is primarily expressed in large neurons (Fig. 3C), serotonin receptor 5HT1F is expressed in a subset of these large cells (Fig. 3D), and MAP4 is expressed in virtually all HVC neurons (Fig. 3E).

### Gene Ontology (GO) identifies cellular targets and processes regulated in HVC

**Figure 4:**
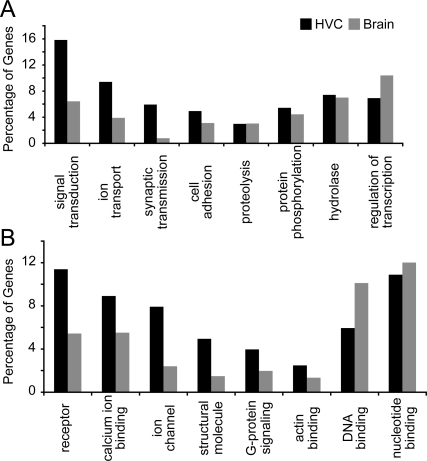
AgBase (http://www.agbase.msstate.edu/) was used to assign GO terms (www.geneontology.org) to as many genes as possible from the FDR <0.05 marker list, and to a set of a randomly sampled 1415 ESTIMA Unigenes expressed in zebra finch brain (see text for details). (A and B) Bar graphs show the percentages of genes that were categorized as involved in a specific biological process (A) or activity (B) that minimally describes 3% of the genes in ESTIMA (Grey columns) or in the FDR<0.05 marker lists (Black columns). For each category, black columns with higher or lower percentages than the corresponding grey column are considered over- and under-represented in HVC, respectively. Because some genes are represented by more than one category, percentages in both graphs sum to more than 100%.

We used GO analysis to identify biological processes and/or molecular functions that are over- or under-represented among HVC regulated genes (i.e., those differentially expressed between HVC and Shelf) as compared to a larger list representing a broader universe of genes expressed in the songbird brain (i.e. a subset of the ESTIMA collection of brain-expressed genes). Because GO comparisons do not take into account the magnitude or direction of gene regulation (up or down), all HVC markers were included in the analysis. Several GO categories were over-represented among HVC markers, including signal transduction, ion transport, and synaptic transmission (Fig. 4A). Accordingly, >40% of the products of HVC markers (as compared to 21% for the broader brain collection) localize to the plasma membrane, and a majority have receptor, ion channel, calcium ion binding, and G-protein signaling activities (Fig. 4B). Genes involved in cell adhesion (Fig. 4A), including several with actin-binding and structural molecule activity (Fig. 4B) were also over-represented. In contrast, genes primarily localized to the nucleus, including genes with DNA binding and transcriptional activity (Fig. 4B) were under-represented. General biochemical processes such as proteolysis, phosphorylation, and hydrolysis had large numbers of hits, comparable to the general brain sample.

### Specific pathways and cellular functions are targets of regulation in HVC

As presented next, we were able to classify the markers in our primary list into categories representing specific genetic, biochemical and cellular functions (Tables 2–7). A summary of these markers is presented in Table S2 and literature citations supporting their functional classification can be found in References S1. The analysis of these pathways was complemented by examining our secondary list (Table S2; these genes are indicated in the text with an asterisk), as well as genes that were functionally related to markers on our primary list but that were not differentially expressed or on the array (Table S5).

**Table 2:** Neuronal Cell Structure.

***CELL ADHESION***	
**Adherens**	
**+**	PARD3, PTPRF, RHOB, WASL
***−***	DACT1, DOCK4, LIN7A, PVRL1, PPFIBP1
**Tight Junctions:**	
**+**	PARD3
***−***	EPB41L2, PRKCD
**ECM, focal adhesion:**	
**+**	COL21A1, COL4A2, DCN, FNTM2, ITGAX, THBS4, TNR
***−***	ADAMTS8, CPNE2, CPNE8, PDGFRA, RELN
**Other Related:**	
**+**	ADAM23, SCUBE1, SCUBE1/2
***−***	MEGF6, NRXN3
***NEURITES***, ***DENDRITES***, ***AND AXONS***	
**Axon Guidance:**	
**+**	NRP1, PDZRN3, PLXNA4
***−***	PLXNA1, SEMA3A, SEMA6A, UNC5C
**Neurite Outgrowth and Extension:**	
**+**	DOK4, MCF2, NDRG4, S100B, STMN3, TNR
***−***	BMPR2, GAS7, LPPR4, PLD1, RASGRF1
***CYTOSKELETON ORGANIZATION***	
**Ankyrin, Spectrin, Actin, and Formin:**	
**+**	ANK1, INF2, ITGAX, WASL
***−***	ANK3, CAP2, CLMN, EPB41L2, FNBP1, KLHL2, MARCKS, MTPN, MYO1B
**Microtubules:**	
**+**	MAP1B, MAP4, S100B, STMN3
***−***	EML5, MAP7, PHLDB2, TUBGCP5
**Neurofilaments:**	
**+**	NEFH, NEFM, NEFL
***−***	none

Tentative gene identifications are underlined (see methods for details).

**Table 3 pone-0003440-t003:** Neurotransmission and Excitability.

***NEUROTRANSMISSION***	
**Neurotransmitters and Receptors:**	
**+**	CHRNA5, CHRNA7, DIP2A, GABRE, HTR1F, PDZRN3, PRKCD, RGS4, SLC38A10
***−***	GLRA2, GRIA4, GRIK2, GRM1, GRM5, HRH3, PLD1, SLC8A1, SLC32A1
**Neuropeptides and Receptors:**	
**+**	CALCR, CHGB, CRHBP, NPY2R, NTS, UTS2B
***−***	CCK, MARCKS, TAC1
**Synaptic Vesicle-related:**	
**+**	CADPS2, SV2B, SYNJ1
***−***	SYT10
***EXCITABILITY***	
**Sodium and Potassium Channels:**	
**+**	DPP6, DPP10, KCNA1, ATP1B4
***−***	ANK3, KCNIP1, KCNC2, KCND2, KCNF1, KCNK5, KCNK10, SCN3B, SHC3

Tentative gene identifications are underlined (see methods for details).

**Table 4 pone-0003440-t004:** Cell Signaling.

***CALCIUM-RELATED***	
**Calcium Channels:**	
**+**	none
***−***	CACNA1G, CACNG4
**Calcium-binding and Signaling:**	
**+**	ANXA6, CABP1, CADPS2, CAMK1D, PVALB, RCAN2, RGS12, S100B, SLC8A1, STC2,THBS4
***−***	CAMK2D, CPNE2, CPNE8, GPR98, HPCAL1, KCNIP1
***PHOSPHORYLATION***	
**Kinases, Phosphatases:**	
**+**	CAMK1D, MAPK11, MGC42105, PPP4R2, RP6-213H19.1, STK11IP
***−***	ARPP21, CAMK2D, LPPR4, PRKAR2B, PRKCD, PTPRZ1, SNRK
**Phosphorylation Related:**	
**+**	PDE8
***−***	PLD1, PTER
***G-PROTEIN RELATED***	
**G-coupled Receptors:**	
**+**	CALCR, HTR1F, NPY2R, PTGER4, RGS4
***−***	GRM1, GRM5, GPR173, GPR177, GPR98, HRH3, RGS12, RGS16
**Small GTPases:**	
**+**	MCF2, RAB39, RASGRP1, RGS4, RHOB, STARD13
***−***	ARL5B, DOCK4, RAB36, RASD2, RASGRF1, RASL11A, RASL12, RGS12, RGS16

Tentative gene identifications are underlined (see methods for details).

**Table 5 pone-0003440-t005:** Cell Growth and Apoptosis.

***CELL GROWTH***	
**Proliferation and Cell Cycle Progression:**	
**+**	AIM1, GBAS, LMO2, LMO3, MCF2, MAP4, NETO1, QSOX1, RP6-213H19.1, SYF2, UBE2E1
***−***	ENOX1, FAM84A, GAS7, KLHL2, RASL11A, RFTN1, TMEM158, TRIB2
**TGF-beta Superfamily:**	
**+**	DCN, FST, NBL1, PDZRN3, SAP30L, THBS4, ZEB2
***−***	BMPR2, CHST11, FAM5C, PRKCD, RSPO3, ZNF423
**PDGF-related:**	
**+**	No hits
***−***	NOP5/NOP58, PDGFRA, PRR5, RGS12
***NEURONAL STABILITY***	
**Migration, Differentiation, and Survival**	
**+**	GPC3, GPI, RGS12, ST8SIA4, TBR1, TNR
***−***	ARHGDIB, ASTN1, RELN, RFTN1
***APOPTOSIS***	
**Apoptosis and/or P53 Related.**	
**+**	AIFM1, RP6-213H19.1, STC2
***−***	ARHGDIB, BAI3, FAM152A, PRKCD, RPRML, SCN3B, SHC1, SHC3, SNRK, TNFAIP8L3, TRP53i11, UBE2D3

Tentative gene identifications are underlined (see methods for details).

**Table 6 pone-0003440-t006:** Gene Expression.

***GENE EXPRESSION REGULATION***	
**Histone Deacetylase/Chromatin Remodeling/Nucleolar Organization**	
**+**	ENDOGL1, PPP4R2, SAP30L
**−**	CNOT6, NAP1L4, NOL4, NOP5/NOP58, SNRK, TCOF1
**Transcription Factors and Regulators:**	
**+**	CAMTA1, DIP2A, FOSL2, NDRG4, SOX6, SYF2, THOC4, TBR1
**−**	CREB1, HMG2L1, HOPX, MEIS1, MYCN, NEUROD1, PCBP3, TOX2, ZNF423
**Translation Factors:**	
**+**	PRKRIP1, RPL18A
**−**	PAIP1
***STEROIDS***	
**Corticoid-related**	
**+**	CRHBP, NR3C2
**−**	none
**Androgen:**	
**+**	RGS4
**−**	EPB41L2, TMEPAI
**Estrogen:**	
**+**	C1orf34, QSOX6, SNCG, STC2, FASN
***−***	CACNA1G, CYP19A1, CYP1B1, PRKCD, RASL11A
**Cholesterol Metabolism, Other Steroids:**	
**+**	none
−	ABCA1, DHRS2
***RETINOIC ACID***	
**Retinoic Acid Signaling**	
**+**	ALDH1A2, ANXA6, LMO2, LMO3, NBL1, NDRG4, PLD1, RHOB, RP6-213H19.1, SACS, TNR
**−**	AQP1, CAMK2D, CLMN, FAM5C, MARCKS, MEIS1, MYCN, PDGFRA, PLD1, UBE2D3

Tentative gene identifications are underlined (see methods for details).

**Table 7:** Other Functions.

***ANGIOGENESIS, AND BLOOD BRAIN BARRIER (BBB) FORMATION***	
**Angiogenesis:**	
*+*	AHNAK2, COL4A2, NRP1, RCAN2
*−*	ADAMTS8, ANTXR2, BAI3
**BBB Formation:**	
*+*	AHNAK2, ABCC5, CD59
*−*	none
***METALLOPEPTIDASES, CARBOXYPEPTIDASES, PROTEASES***	
**Metallopeptidases, Carboxypeptidases, Proteases**	
*+*	ADAM23, CPM, LGMN
*−*	ADAMTS8, MMEL1, TIMP3, TLL1, USP46

Tentative gene identifications are underlined (see methods for details).

#### 1. Cell adhesion, neurite outgrowth and cytoskeletal organization (Table 2)

Genes that have been linked to the regulation of cell structure, including cellular adhesion, neurite outgrowth and extension, and cytoskeletal organization represented nearly 25% of our primary list. Among these, we detected a small cluster involved in tight junctions and a larger cluster related to cadherin-catenins, which are major components of adherens junctions in neuronal and epithelial tissues. The latter included regulators of catenins (PTPRF, PPFIBP, DACT1, LIN7A), modulators of adherens interactions with the cytoskeleton (RHOB, WASL, DOCK4), as well as a cadherin (CDH1*) and its downstream target alpha-catenin 1 (CTNNA1*; CTNNA2 was not differential), which were all HVC-enriched. In contrast, nectin (PVRL1), a non-cadherin component of adherens, was low in HVC, as was SSX2IP*, a protein that interacts with afadin in the nectin pathway.

Several markers represented components of the extracellular matrix (ECM) or their receptors (e.g. integrins and receptor tyrosine kinases), known to be involved in the formation and maintenance of focal adhesions. This included the HVC-enriched collagens (COL21A1, COL4A2, COL2A1*, COL6A1*, COL10A1* and COL12A1*), decorin (DCN), tenascin (TNR), thrombospondin (THBS4), and a fibronectin III domain-containing protein (FNTM2), all of which are ECM components, as well as an alpha-integrin (ITGAX). In contrast, PDGFRA, RELN, and several modulators of integrin binding (CPNE2/8, and ADAMTS8) had decreased expression in HVC. Seven other collagens as well as most of the integrins were not differential. Other markers encoded proteins that participate in broader aspects of cell adhesion, including MEGF6, SCUBE1/2, SCUBE1, NRXN3, which all possess epidermal growth factor-like domains, and ADAM23, a disintegrin/metallopeptidase with thrombospondin-1-like activity that may bind integrins.

**Figure 5:**
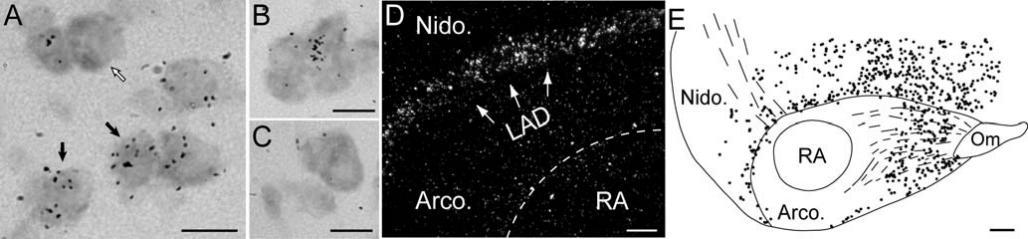
(A–C) Bright-field high power Nissl stained views of NRP1 emulsion autoradiography at the level of HVC. Black arrowheads indicate examples of small cells with high levels of emulsion grains (A), white arrows point to larger neurons with no detectable expression (see also C). (D) Low power dark-field autoradiography for SEMA3A in a parasagittal section at the level of RA. Note intensely labeled cells that align along the lamina arcopallius dorsalis (LAD). (E) Camera lucida drawing of the ventral nidopallium and arcopallium from the section shown in D. Individual cells expressing SEMA3A are represented by solid circles. For anatomical abbreviations see text. Scalebars: 10 µm in A–C; 100 µM in D; 1 mm in E.

A significant cluster has been linked to axonal guidance. This included the upregulated neuropilin (NRP1) and plexin-A4 (PLXNA4), which together comprise the receptor for semaphorins 3A and 6A (SEMA3A and SEMA6A), and a PDZ-domain containing ring-finger protein (PDZRN3) that interacts with semaphorins, as well as the downregulated PLXNA1, PLXNB1* (PLXNA2 was not differential), and UNC5C, the latter a receptor for netrins, which are non-semaphorin mediators of axonal guidance (other plexins and semaphorins were not differential). *In situ* hybridization confirmed that both NRP1 and PLXNA4 are enriched in HVC, and revealed that NRP1 is primarily expressed in clusters of small (Fig. 5A–B), but not large (Fig. 5C) HVC neurons that resemble either RA-projecting cells or interneurons [37]. In contrast, the ligand SEMA3A was confirmed downregulated in HVC and its targets RA and area X (not shown), but was highly enriched in a band of large cells adjacent to the dorsal arcopallial lamina and intercalated with nidopallial axonal bundles entering the arcopallium (Fig. 5D and E).

Finally, we found several clusters containing genes implicated in various aspects of neurite outgrowth, extension, and in the initial stages of the formation of axonal processes, as well as cytoskeletal organization. For example, several genes involved in promoting (DOK4, MCF2, NDRG4) or inhibiting (STMN3, TNR and S100B) neurite extension/outgrowth were higher in HVC than in the Shelf, while the genes in this category that were lower in HVC are involved in promoting neurite outgrowth and/or dendritogenesis. Another large cluster consisted of markers implicated in cytoskeletal organization through interactions with actin (CLMN, CAP2, KLHL2, ITGAX, MARCKS, MYO1B, WASL), spectrin (EPB41L2), ankyrin (ANK1, ANK3, MTPN), and formins (FNBP1, INF2), most of which had decreased expression in HVC. In addition, the microtubule-associated proteins MAP1B and MAP4, a microtubule destabilizing factor (STMN3), and an inhibitor of microtubule assembly (S100B) had enriched expression in HVC, while some microtubule-stabilizing proteins (MAP7 and PHLDB2), a regulator of microtubule nucleation (TUBGCP5), and a member of the echinoderm microtubule-associated protein family (EML5) were lower in HVC.

#### 2. Neuronal Transmission and Excitability (Table 3)

Our microarray data confirmed the lower expression in HVC of several members of the ionotropic and metabotropic glutamate receptor family (GRIA4, GRIK2, GRM1, GRM5) and the higher expression of a splice variant of glutamate receptor interacting protein 1 (DIP2A), which has known glutamate receptor binding activity. We also detected a higher expression in HVC of a fast-gating epsilon subunit of the GABAA receptor (GABRE) and a putative (MGC15523) vesicular transporter of GABA, as well as the lower expression of a known GABA transporter (SLC32A2). The glycine receptor subunit alpha-2 (GLRA2; Fig. 2D, right panel) had very low expression in HVC, but was significantly expressed in Shelf.

**Figure 6:**
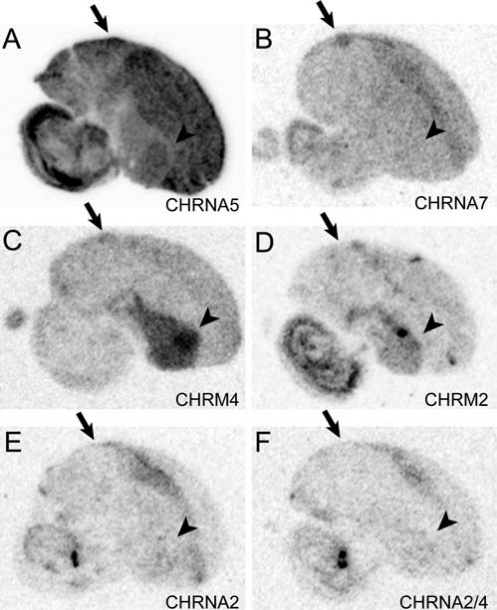
(A–F) Phosphorimage autoradiograms showing the patterns of expression for nicotinic (in A, B and E, F) and muscarinic (in C, D) acetylcholine receptor subunits in sections hybridized with antisense probes direct against various receptor subunits. See text for additional details and gene name abbreviations. Arrows indicate the locations of nucleus HVC in each as identified under bright-field illumination in an adjacent Nissl stained section, arrowheads indicate the location of area X. Scale bar: 1 mm.

Several cholinergic receptor subunits had high expression in HVC, including both nicotinic (CHRNA5, CHRNA7) and muscarinic (CHRM4*) family members, as well as PDZRN3, a ring finger protein implicated in acetylcholine receptor clustering. In contrast, other members of the cholinergic receptor family were not differential (CHRNA2, CHRNA9, a clone with similarity to both CHRNA2 and CHRNA4, and CHRM2) or not on the array (CHRNA1/3/6, CHRNA8, which is unique to birds, nicotinic beta-subunits CHRNB1/2/3, and CHRM1/3/5). *In situ* analysis verified the enrichment of CHRNA5, CHRNA7 and CHRM4* in HVC (Fig. 6A–C), and confirmed the lack of differential expression for CHRM2, CHRNA2 and CHRNA2/4 (Fig. 6D–F).

We detected the HVC enrichment of several components related to serotonin-mediated signaling. While serotonin receptor subunits HTR1B and HTR7A were not differential, HTR1F and a regulator of G-protein signaling (RGS4) that mediates a serotonergic down-regulation of NMDA-receptors were enriched in HVC. *In situ* hybridization indicated that HTR1F was predominantly expressed in a subset of large cells (Fig. 3C). Finally, the G-protein coupled histamine receptor (HRH3) was low in HVC, suggesting a downregulation of this modulatory aminergic pathway.

We detected several markers related to peptidergic transmission, including the peptide secretory granule protein CHGB. In particular, the precursor of neurotensin/neuromedin N (NTS), a peptide whose release is mediated by MARCKS (which was downregulated), as well as UTS2B, a peptide with potent vasoconstrictor activity, were enriched in HVC. Several peptide receptors (CALCR, NPY2R, CRHBP) were also higher in HVC, and this contrasted with CCK and TAC1, which were lower in HVC. Another small cluster was more generally related to synaptic vesicle physiology.

Several markers represented structural or regulatory components of sodium and potassium channels. Our analysis revealed that multiple K-channel subfamily members are often co-regulated at the exclusion of other members or subfamilies that would be predicted to confer similar properties. For example, among fast-inactivating subunits, KCNA1 and its accessory subunit KCNAB1* were higher in HVC (KCNA4 and KCNA6/7 were not differential and other members were not on the array). In contrast, KCND2 was lower in HVC, and two dipeptidyl-peptidases (DPP6 and DPP10) that can act as KCND-type inhibitory accessory subunits were enriched in HVC. Among delayed-rectifier subunits, KCNC1* and KCNC3* were up in HVC, whereas KCNC2 and its modulatory subunit (KCNIP1) were down. Although KCNB delayed rectifiers were not on the array, they also appeared to be down in HVC based on the relative lower expression of cooperative subunits (KCNF1, KCNG1*, KCNS2*), and an enrichment of an inhibitory subunit (KCNV1*) of this subfamily. Finally, two open-rectifying leak channels (KCNK5, KCNK10), a sodium channel modulatory subunit (SCN3B), and ANK3, a member of the ankyrin family that can cluster sodium or potassium channels, all shared a lower expression in HVC.

#### 3. Calcium- and G-protein related signaling and phosphorylation (Table 4)

Several markers have been linked to calcium entry and binding, and the activation of signaling and phosphorylation cascades. We observed that calcium-channel related genes generally had lower expression in HVC, including a T-type low-voltage activated subunit (CACNA1G), an L-type channel (CACNA1D*), and an accessory subunit (CACNG4) known to associate with L-type channels. Other subclusters could be classified as calcium-dependent, capable of binding calcium, or calmodulin-like in their activity. Among these, we detected an HVC enrichment of genes involved in calcium-dependent liposome binding (ANXA6) and dense-core vesicle exocytosis (CADPS2; Fig. 2C middle), and lower expression of genes involved in mediating ECM:integrin interactions (CPNE2 and 8). Genes with known high calcium affinity and/or sensitivity included a regulator of calcineurin 2 (RCAN2), a gene involved in cell adhesion (thrombospondin; THBS4), and a structural protein that contains an EF-hand calcium-binding motif (S100B), which were enriched in HVC, and a G-protein coupled receptor (GPR98), a hippocalcin-like protein (HPCAL1), and an A-type potassium channel regulatory subunit (KCNIP1), which had lower expression in HVC. Other markers of enriched expression in HVC included a calcium-binding protein (CABP1) that complexes with L-type calcium channels, two calmodulin delta chains (CAMK1D; CAMK2D), and a previously described calmodulin-like marker (parvalbumin; PVALB).

Several markers were related to protein phosphorylation/dephosphorylation. Besides calcium-sensitive genes, these included a mitogen-activated protein kinase (MAPK11), serine/threonine kinases (MGC42105, PRKCD, PTPRZ1, RP6-213H19.1, SNRK), a cAMP-dependent protein kinase (PRKAR2B), protein phosphatases (ARPP21, LPPR4, PPP4R2), and phosphorylated proteins with phospholipase or phospho-(di/tri)-esterase activity (PLD1, PTER, PDE8). Finally, many markers were related to G-protein signaling. Specifically, G-protein coupled receptors for prostaglandin (PTGER4), and for several neurotransmitters (HTR1F, CHRM4*, NPY2R, CALCR) were enriched in HVC, whereas those for histamine (HRH3) and glutamate (GRM1 and GRM5) were low in HVC, as were other members of this gene family (GPR98, GPR173, and GPR177). We also identified an enriched modulator of small GTPase activity (MCF2) along with several differentially-expressed members of the Ras superfamily of small GTPases, suggesting a prominent role for GTP-related signaling in HVC.

#### 4. Cell Growth and proliferation, neuronal stability, and apoptosis (Table 5)

Nearly a quarter of the HVC markers were linked to cell proliferation, migration, differentiation and survival. Specifically, one large cluster is involved in cell proliferation and cycle progression, including HVC-enriched genes that mediate non-p53 tumor suppression (AIM1, GBAS, NETO1, UBE2E1; LMO2, LMO3), interact with cyclin D1 (SYF2), or generally relate to cell proliferation (QSCN6), as well as a variety of markers of low expression associated with tumor suppression, growth arrest and/or proliferation.

A second large cluster is related to the TGF-beta signaling pathway. Specifically, while a single component of the TGF-beta receptor (TGFBR2*) was enriched in HVC, several inhibitors of TGF (DCN, THSB4, LTBP1*) or activin (FST) signaling were also enriched. Several positive mediators of signaling had lower expression in HVC, including an activator of TGF-beta signaling (RSPO3) and an activin receptor component (ACVR1*; no activins were on the array). Other TGF-beta-related genes were not differential, including the only TGF-beta on the array (TGFB3), as well as a TGF-beta inducible nuclear protein (TINP1), a TGF-beta induced apoptosis protein (FAM130A2), an activin A receptor (ACVR2A), and downstream regulatory proteins (SMAD1, 2, 6 and 9). A subset of TGF-beta related genes belonged to the bone morphogenetic protein (BMP) pathway, including the HVC-enriched BMP receptor antagonists (NBL1, ZEB2), and a BMPR2 interactant (PDZRN3), as well as a BMP receptor (BMPR2) and two mediators of BMP activation (CHST11, and ZNF423), and the BMP target FAM5C, which were low in HVC. The only BMP on the array (GDF3) was not differential, the BMP signaling inhibitor NOG was highly expressed in HVC but not differential, and other BMP receptors (BMPR1A, BMPR1B and AMHR2) were not on the array. A smaller cluster of genes with lower expression in HVC included the platelet-derived growth factor receptor A (PDGFRA), regulators of the PDGF-pathway (PRR5 and RGS12), and PDGF-inducible NOP5/NOP58.

Other markers have been specifically linked to apoptosis and/or p53 tumor-suppressor function. Among HVC-enriched genes were an apoptosis-inducing factor (AIFM1, a.k.a. programmed cell death 8, or PDCD8; the PDCDs 2, 4, 5, and 6 were not differential), a kinase (RP6-213H19.1) that interacts with PDCD10, and a secreted glycoprotein (STC2) that is induced by estrogen and protective against apoptosis. However, the majority of markers related to apoptosis had lower expression in HVC, including modulators of angiogenesis (BAI3, SHC1, SHC3, SNRK, FAM152A, and PRKCD), a mediator of TNF-induced apoptosis (TNFAIP8L3), a regulator of cell protection against apoptosis (TRAIP*) and genes that are mediators of p53 activation (UBE2D3, RPRML) or induced by p53 (SCN3B, TRP53i11, and the growth arrest factor GADD45G*). Another important mediator of apoptosis (CASP3) and a neuroprotective gene (BFAR) were not differential.

Some HVC markers have been linked to cell migration, differentiation and survival, and play important roles in the maturation and integration of post-mitotic cells into mature circuitry, and patterning during development. These included a cell surface heparan sulfate proteoglycan (GPC3), a neurotrophic factor (GPI), and genes involved in the regulation of adhesion and ECM formation during neural development (ST8SIA4, TBR1, TNR), which were enriched in HVC, while known mediators of migration (ASTN1, RELN, RFTN1) were lower in HVC.

#### 5. Gene Expression (Table 6)

A number of HVC markers could be classified as being related broadly to gene expression regulation. For example, two histone acetylation/deacetylation proteins (PPP4R2, SAPL30), a chaperone complex protein related to chromatin binding (THOC4), an endonuclease (ENDOGL1) and an RNA-splicing factor (SYF2) were all enriched in HVC, as were genes involved in protein translation initiation and elongation (PRKRIP, RPL18A). In contrast, nucleolar proteins (TCOF1, NOP5/NOP58, and NOL4), genes related to histone/nucleosome assembly and chromatin remodeling (SNRK, NAP1L4), and a poly(A) binding protein (PAIP1) had lower expression in HVC. Several other markers had DNA binding and/or transcription factor (TF) activity, including a calmodulin-binding TF (CAMTA1), a repressor of TF activity (DIP2A), a mineralocorticoid receptor (NR3C2), a member of the myc family (MYCN), and two homeobox genes (HOPX, and MEIS1).

Several markers are involved in the metabolism and/or actions of steroids. For example, a corticotropin-releasing hormone binding protein (CRHBP; Fig. 2B), and a mineralocorticoid receptor (NR3C2) were enriched in HVC, as were three genes with known androgen-related action, namely, an androgen-sensitive regulator of G-proteins (RGS4), which was enriched in HVC, and an androgen-induced prostate cancer RNA (TMEPAI) and an androgen regulated FERM domain containing protein (EPB41L2), which had lower expression in HVC. Among estrogen-related genes, we observed a lower expression in HVC of CYP1B1, which metabolizes 17-beta-estradiol, and of CYP19A1 (a.k.a. P450 aromatase), the enzyme that converts testosterone into estrogen. Others genes in this cluster were targets of estrogen action (e.g. T-type calcium channel subunit; CACNA1G), capable of binding estrogen receptor alpha (FASN, SNCG), or involved in tumor suppression (PRKCD, RASL11A). Finally, two genes involved in cholesterol/lipid metabolism (ABCA1, DHRS2) had lower expression in HVC.

Finally, we confirmed a strong HVC enrichment of zRalDH (a.k.a. ALDH1A2) and identified several large clusters of markers that have been implicated in broad aspects of retinoic acid metabolism and/or function, or that have been shown to be regulated by retinoic acid in other systems. This included markers related to gene transcription (NDRG4 and MEIS1, GTF2H1*, JUN*, and CBX3*), growth suppression and/or apoptosis (LMO2, LMO3, RHOB, NBL1, FAM5C, UBE2D3) retinoic acid metabolism (ANXA6, HSD17B11/13* - the latter consistent with the in situ pattern in [29]), or known regulated genes with undefined functions (RAI1*, RAI14*, DBC1* - the latter confirmed by in situs in [29]). We also found that several markers are part of specific signaling pathways that have been implicated in various aspects of neuronal development and differentiation, including: (1) wnt signaling (through the HVC-enriched antagonists FRZB* and SFRP2*, and the low expression of CAMK2D), (2) notch signaling (through the low expression of the receptor JAG1*), (3) the BMP pathway (through an HVC enrichment of the suppressor NBL1 and the low expression of the target FAM5C, consistent with low expression of the receptor BMPR2), (4) the MYCN pathway (through an HVC enrichment of MYC-suppressed genes NDRG1* and 4, consistent with low expression of MYCN itself), (5) cell adhesion (through enrichment of the ECM component TNR, the adherens modulator RHOB, and the axonal guidance mediator CXCR4*).

#### 6. Other Functions (Table 7)

One cluster was related to angiogenesis and blood brain barrier formation. While all of the enriched genes were positive regulators of angiogenesis, genes with anti-angiogenic activity had lower expression in HVC, including ANTRX2, a positive regulator of capillary morphogenesis, and two brain-specific angiogenesis inhibitors (BAI3). A second cluster represents protein-hydrolyzing endopeptidases, and includes members of the metalloproteinase family, a membrane-bound arginine/lysine carboxypeptidase (CPM), and legumain (LGMN), a cysteine protease.

## Discussion

Our results provide a comprehensive classification of more than 280 genes from our primary HVC marker list that are related to a wide variety of genetic, biochemical and cellular functions as presented in Tables 2–7. This analysis was complemented by a careful review of genes from our secondary lists (Tables S2 and S5) that in the majority of cases provided additional support for our primary classification, and aided in our interpretation of relationships between biochemical pathways and the intrinsic properties of HVC. A primary goal of our study was to identify molecular specializations of the adult nidopallium that constitute neurochemical markers of nucleus HVC. Thus, our data represent a comprehensive survey of the intrinsic physiological properties of the adult HVC. However, since the primary functions of HVC are in the production and memorization of learned song, it seems reasonable to suggest that many of these neurochemical specializations may also bear relationships to these processes, although clearly functional follow-up experiments will be needed to establish such relationships. The fact that we used unstimulated quiet birds for our screening minimized the chances of including genes whose expression depends on neuronal activation during singing or hearing. Thus, the markers we have identified represent bona fide markers whose differential expression does not require singing or hearing activities. We note, however, that these markers may still represent cellular functions that could in principle modulate the processes of singing and/or learning how to sing. We also note that while the microarray data do not allow us to conclude whether a given differentially expressed marker results from regulation in the HVC or Shelf region, all the genes analyzed by *in situs* clearly differentiate HVC from the Shelf, while none differentiate the Shelf from the rest of the nidopallium. These observations suggest that the properties of the Shelf region are mostly reflective of the nidopallium at large. More importantly, we infer that the markers we have identified are mostly the result of differential (up- or down-) regulation within HVC, and that combined they represent a specific program of gene regulation that differentiates HVC from the rest of the avian nidopallium. Below we discuss how our key results have led to the formulation of novel hypotheses about neurochemical and molecular processes that may be regulatory targets related to the maturation and/or function of the vocal-motor circuitry, and thus potentially affect song learning and/or production.

### Cellular excitability, Intracellular signaling, and Cell-to-cell communication are HVC

Our GO analysis revealed that the products of HVC markers are almost twice as likely to be localized to the plasma membrane as compared to a broader collection of brain-expressed cDNAs. The fact that the majority of these markers have receptor, ion channel, calcium ion binding, and G-protein signaling activities (Fig. 4B) further suggests that HVC neurons tightly regulate features of cellular excitability, intracellular signaling, and cell-to-cell communication. Similarly, the over-representation of genes involved in cell adhesion (Fig. 4A), including several with actin-binding and structural molecule activity (Fig. 4B), suggests a particularly important role for the regulation of cellular, neuritic, and synaptic morphology through cell-cell and cell-extracellular matrix interactions in HVC. In contrast, the under-representation of genes primarily localized to the nucleus, including genes with DNA binding and transcriptional activity (Fig. 4B), is consistent with previous reports that the HVC of non-singing birds expresses low levels of activity-regulated transcription factors (e.g. *zenk* and *c-fos*). In sum, the GO analysis pointed to several regulatory themes that seem to be important specializations of HVC, as discussed in more detail in the following sections.

### Expression analysis of HVC Markers

The discovery of bona fide markers based on the comparison of HVC vs. the underlying nidopallial Shelf provides important support for our rationale that HVC constitutes a molecular specialization of the nidopallium that is the product of specific programs of gene regulation. Furthermore, because our microarray comparison was not to the whole brain, we were able to identify markers that are also expressed in other song nuclei and brain subdivisions in various combinations (Figure 2 and Table S4). Thus, some molecular specializations of HVC potentially reflect properties that are common among subsets of song nuclei. For example, similar to zRalDH, an enrichment in HVC and LMAN may reflect a nidopallial characteristic that is absent from arcopallial and/or striatal nuclei (e.g. the local synthesis of retinoic acid). In contrast, a shared enrichment in HVC and striatal area X might indicate a possible involvement in the establishment and/or function of the HVC-to-X projection system, and a shared enrichment in HVC, RA and LMAN could suggest a molecular pathway related to pallial but not striatal function. We also identified some genes (e.g. CADPS2, NTS and S100B) that showed enrichment in distinct pallial areas such as primary auditory field L2a and somatosensory nucleus basorostralis, suggesting that some molecular properties of HVC may be shared with more general telencephalic subdivisions. Future comparisons between HVC and other song nuclei and/or pallial regions may reveal additional information about possible shared regulatory programs in the avian brain, as well as more specific clues about the molecular origins of the song system.

In contrast to shared markers, the markers found to be exclusive to HVC may reflect functional properties unique to HVC. MUSTN1 (Fig. 2B, left), for example, is associated with cellular proliferation and differentiation in bone tissue [38], and might play similar roles in the development and/or growth of newly-born neurons that continuously arrive in HVC, even in adults. Interestingly, in a preliminary study with Anna's hummingbirds (wild-caught and kept in isolation for about 2–3 hr prior to sacrifice to minimize singing-induced gene expression; Lovell and Mello, unpublished observ.), MUSTN1 is also a specific marker of VLN (vocal nucleus of the lateral nidopallium), the presumed hummingbird analog of HVC (not shown). Thus, some molecular specializations of a nucleus thought to be independently derived for song learning and production may be conserved across avian vocal learning lineages. Further tests of whether MUSTN1 and other genes are general markers of HVC-like nuclei in avian vocal learners are currently underway.

Emulsion autoradiography revealed that some HVC markers have distinct cellular distributions, suggesting that our markers may label different cell types in HVC and provide potentially important clues about cellular specializations within the song system. Of particular interest, our results confirm that at least one HVC-enriched serotonin receptor subunit (5HT1F) is expressed in a subset of large HVC neurons, possibly corresponding to X-projecting neurons. HTR1B and HTR7A, which were not differential based on microarray analysis might also be present in HVC, but this will required further confirmation. Notably, there is precedence for serotonergic modulation in HVC. Specifically, serotonin has been shown to hyperpolarize the larger Type I HVC neurons, and to a lesser extent Type II neurons (thought to be respectively X- and RA-projecting neurons; [37]). Our results support these observations, and while cell specificity will need to be directly confirmed by tract-tracing and/or double-labeling methods, our initial results provide the identification of a possible molecular mechanism mediating HVC modulation by serotonin. Importantly, since a1 serotonergic receptor subunits are quite sensitive to a variety of agonists/antagonists (e.g. smatriptan, methiothepin), these reagents may provide a new and useful set of tools for manipulating the song system during song production and/or learning. In contrast, other markers like NRP1 were found to be more closely associated with small, possibly RA-projecting neurons (Fig. 5A). Intriguingly, CRHBP appears to be a unique marker of a previously undescribed neurochemically distinct HVC subpopulation (Fig 3B). The distribution and density of CRHBP-containing cells resembles that of newly-formed RA-projecting neurons [39]–[41], an intriguing possibility requiring further investigation. Continuing studies of cellular localization should further contribute to a broader understanding of molecular and cellular specializations of the song system.

### Cell adhesion, neurite outgrowth and cytoskeletal organization

**Figure 7:**
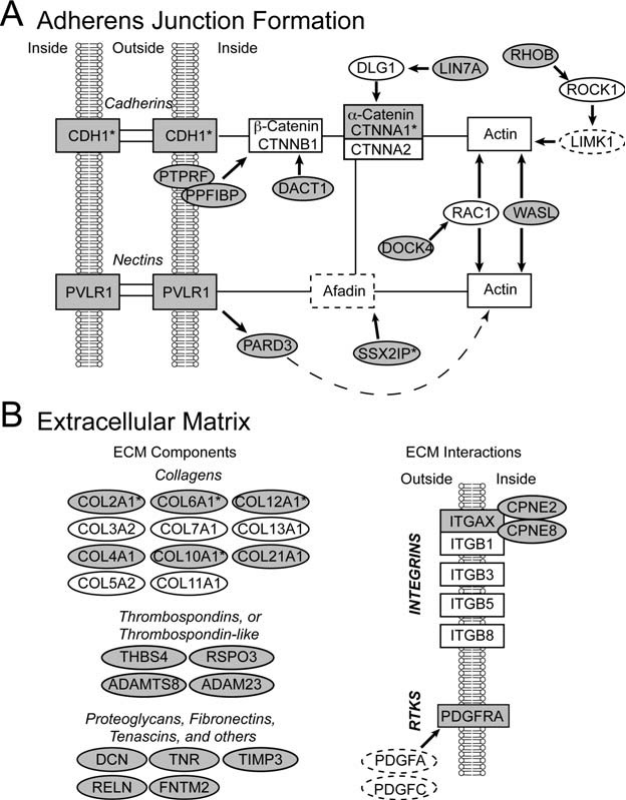
Networks describing the involvement of HVC markers in the formation of adherens junctions (A) and in the mediation of focal cell adhesions to the extracellular matrix (ECM) (B). Gene abbreviations are in the text and Tables S1 and S3. Rectangles constitute central nodes with specific receptor and/or structural properties, genes in ovals play a primarily regulatory role. Arrows indicate a positive direct interaction, solid lines depict bi-directional protein-protein interactions. Not all connections and nodes are included; genes not required for network assembly, or not present on the arrays were omitted for clarity. Grey elements represent differentially expressed genes from our primary or secondary (asterisks), white symbols indicate genes that were either not differential (solid), or not on the array (dashed).

The large number of markers revealed by our bioinformatics analysis to be related to cellular adhesion, neurite outgrowth and extension, and cytoskeletal organization suggests that these are major targets of regulation in HVC (Fig. 7; Table 2). Specifically, we observed an overall positive regulation of components related to the formation of adherens junctions (i.e. cadherins, catenins; Fig. 7A), as well as the formation and maintenance of focal adhesions (i.e. collagens, integrins; Fig. 7B), suggesting that cell-cell interactions through adherens junctions (likely mediated by the cadherin-catenin pathway) and collagens/integrins likely constitute specializations of HVC. Since adherens and focal adhesions can provide stabilizing influences, our observations suggest that HVC neurons may be tightly anchored to each other or to the ECM through a variety of junctional complexes. We suggest that the regulation of these genes in HVC may help to maintain connections between existing neurons and glia, and also influence the motility, survival, and differentiation of newly-migrated cells.

Our results also suggest that axon guidance is an important target of regulation in HVC, and that semaphorin-related (not netrin-related) signaling is a major candidate mediator of axon guidance for HVC neurons. Follow-up *in situ* analysis further indicates that while NRP1 is primarily expressed in small neurons (possibly RA-projecting cells), the NRP1/PLXNA4 receptor ligand SEMA3A is generally down-regulated in HVC and its targets (i.e. RA and X). In NRP1/PLXN4 expressing cells, SEMA3A typically facilitates the collapse of growth cones and/or formation of fasciculated axon bundles [42], [43]. Thus, the apparent absence of SEMA3A in RA may play a permissive role for axon extension from HVC's RA-projecting cells, which appear to be NRP1-positive. In contrast, SEMA3A expression is highly enriched in a band of cells that lies adjacent to the dorsal arcopallial lamina and is intercalated with nidopallial axonal bundles entering the arcopallium (Figs. 5D and E). These results suggest a possible role for semaphorin-mediated guidance and/or the fasciculation of new axons extending from HVC to RA. A similar role may be served by SEMA3A-expressing cells that are interspersed with the fibers of the occipito-mesencephalicus tract (OM; Fig. 5E). We are currently conducting a comprehensive developmental analysis that should provide additional clues as to potential roles of semaphorins in axon guidance within the song system.

The overall balance of regulation (decreased expression in HVC) of genes involved in neurite outgrowth and extension, or the initial stages of the formation of axonal processes, suggests a decreased ability of HVC neurons to initiate neurite outgrowth and/or dendritogenesis. Consistent with this possibility, the overall balance of microtubule-associated proteins, destabilizing factors, and inhibitors of microtubule assembly suggest an increased destabilization of microtubule assembly, which is potentially linked to an increase in the stability of cell structure and a decreased ability to initiate neurite extension. Moreover, consistent with previous data [26], all three neurofilaments (NEFH, NEFM, NEFL), which are major structural components of mature neurites and co-assemble as heteropolymers in other systems, are highly enriched in HVC. Such enrichment may favor a general stabilization of existing neuritic processes. Finally, several genes that interact with actin, spectrin, ankyrin, and formins were also differentially expressed, further suggesting that regulation of neuronal process formation and cytoskeletal organization might be important targets of regulation in HVC.

### Neuronal Transmission, Cellular Excitability, and Calcium Signaling

The many markers involved in the assembly, function and/or regulation of neurotransmitter receptors, included several related to peptidergic, aminergic, and cholinergic signaling, and suggested that HVC is a major target of neuromodulation by these pathways (Table 3). The *in situ* analysis confirmed the differential regulation of nicotinic and muscarinic cholinergic receptor subtypes, but also demonstrated a lack of differential expression for several others (Fig. 6), suggesting that subunit specificity may play an important role in HVC cholinergic modulation. Previous reports have shown that HVC receives cholinergic input from the basal forebrain, expresses high levels of acetylcholinesterase [44], [45], and shows responsiveness to auditory input in anesthetized birds that is modulated by stimulation of the basal forebrain or injection of cholinergic agonists [46]. The specific set of cholinergic receptor subunits we have found to be enriched in HVC could serve as part of the mechanism for gating auditory input into HVC. They also provide a molecular basis for the enriched binding for alpha-bungarotoxin (a nicotinic 5/7/9 receptor ligand) and quinuclidinyl benzilate (a general muscarinic antagonist) in HVC [45], [47]. Also consistent with previous data, muscarinic receptors were expressed primarily in the striatum. Although enriched in HVC, CHRM4* was particularly elevated in area X, possibly representing a mediator of cholinergic modulation in the anterior forebrain pathway. In sum, the regulated expression of cholinergic receptors with unique gating properties, conductances, and coupling to second-messenger systems likely help determine the responses of HVC neurons to cholinergic input, and may play important roles in modulating HVC's firing properties under different behavioral states.

We also identified novel candidate peptidergic and glycinergic modulators of HVC. First, the glycinergic receptor subunit GLRA2 has very low expression in HVC, but is significantly expressed in shelf, suggesting a novel role for glycinergic transmission in parts of the adult avian pallium. This is an intriguing possibility, since the avian pallium is thought to share a common origin with the mammalian cortex, and it has been recently confirmed with electrophysiological recordings in awake zebra finches (Lovell et al, unpubl. observ.). We also found that a precursor of neurotensin/neuromedin N (NTS), as well as UTS2B, a peptide with potent vasoconstrictor activity, are both highly enriched in HVC and could represent novel peptides used by HVC neurons themselves. In contrast, several peptide receptors (CALCR, NPY2R, CRHBP) were also expressed, suggesting that their respective peptides calcitonin, neuropeptide Y and CRH may be modulators of HVC.

In general, our results suggest an overall decreased expression of genes in HVC that are related to cellular excitability (Table 3) and an enrichment of genes involved in intracellular calcium regulation (Table 4). For example, delayed-rectifier subunits, KCNC1* and KCNC3*, which are more prevalent in slower firing cerebellar and striatal neurons [48], [49] were enriched, whereas KCNC2, prevalent in fast-spiking mammalian neocortical and hippocampal GABAergic interneurons [50], [51] and its modulatory subunit (KCNIP1) were impoverished. This dichotomy suggests that neurons in HVC may sustain overall lower firing frequencies than the nidopallium as a whole. The low expression of calcium channel subunits, specifically T- and L-types, paired with an upregulation of genes with known affinity for and/or binding of calcium (e.g. CABP1, CAMK1D), suggest that HVC neurons may place a premium on reducing intracellular calcium levels.

In sum, the numerous markers in this category provide important clues about mechanisms that may determine the physiological and synaptic properties of HVC neurons. They also represent novel candidate targets for pharmacological manipulation that may help to address how specific cellular and synaptic properties influence the HVC and the song system. A comprehensive cellular analysis will be important to link the differential expression of specific subunits to individual cell types.

### Cell Growth and proliferation, neuronal stability, and apoptosis

**Figure 8:**
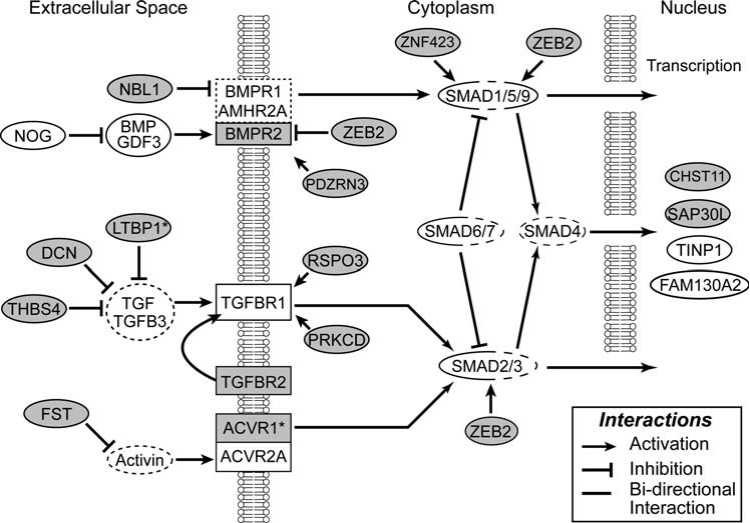
Gene abbreviations are in the text and Tables S1 and S3. Rectangles constitute important nodes in the network that have receptor activities, genes presented in ovals play either a regulatory role, or are products of transcription (shown in the nucleus). Not all connections and nodes are included; genes not required for network assembly, or not present on the arrays were omitted for clarity. Rectangular and oval symbols depicted in grey indicate genes from our primary or secondary lists (asterisks), symbols or fractions of symbols shown in white were either not differentially expressed (solid) or not on our array (dashed). Major cellular compartments are indicated along the top or by the presence of phospholipid membranes.

The dynamics of cell proliferation, migration, differentiation and survival play prominent roles in shaping the anatomical and functional organization of HVC throughout life. Accordingly, nearly 25% of our HVC markers were linked to these processes, including a large cluster of non-p53 related genes involved in cell proliferation and cycle progression that were enriched in HVC, and a set of genes related to tumor suppression and/or proliferation that had low HVC expression (Table 5). A second large cluster was related to TGF-beta (Fig. 8), suggesting that this signaling pathway is a major target of regulation in HVC. Finally, a third cluster has been specifically linked to apoptosis and/or p53 tumor-suppressor function. The low HVC expression of several key apoptosis mediators suggests a general downregulation of apoptosis-related signaling, perhaps favoring increased cell survival. Overall, our data suggest that pathways related to cell growth and proliferation may be largely suppressed in HVC, while those involved in promoting cell survival may be active. These conclusions are consistent with the notion that song in this songbird species is highly stable in adulthood. We suggest that these pathways could be more active during the learning period, when the song system undergoes marked changes in its composition and size. A further test of this correlation will be to investigate the expression of these HVC markers in birds that show fluctuations in song production patterns during adulthood. It is possible that proliferation-related markers localize to the ventricular zone dorsal to HVC, a possible source of newly-formed neurons in HVC that was most likely included in our dissected samples. Alternatively, these markers may relate to the proliferative control of glial and/or endothelial cells. Studies of cellular expression in birds of different ages during song development will be essential to clarify the significance of regulating these pathways in HVC.

Our study also revealed that some HVC markers are linked to cell migration or differentiation, and play important roles in the maturation and integration of post-mitotic cells into mature circuitry, and patterning during development. For example, the extracellular matrix protein TNR plays a unique role in neuronal recruitment out of the migratory stream in the olfactory bulb [52]. Its elevated expression in HVC may thus favor the recruitment of migratory neurons into HVC. ST8SIA4, which is responsible for the sialation of NCAM, is potentially associated with the prominent immunolabeling for poly-sialated NCAM in HVC, thought to be related to the migration of post-mitotic neurons into HVC [53], [54]. On the other hand, the low expression of known migration mediators may reflect an overall low rate of cell migration within HVC. While it is difficult to predict the precise impact of regulating this set of genes, we suggest that the markers we have identified may potentially provide signaling cues help guide that could guide the incorporation and maturation of newly-arrived post-mitotic neurons within HVC.

### Gene Expression

Based on GO analysis, genes associated with cell nuclei, including those encoding factors with DNA-binding and transcriptional activity (Fig. 4), were generally under-represented in the list of HVC markers compared to broader collection of brain-expressed genes. This appears consistent with previous reports that the HVC of non-singing birds expresses low levels of activity-regulated transcription factors (e.g. *zenk* and *c-fos*), and with a recent microarray study that found that 12 out of 32 (∼38%) confirmed singing-induced HVC markers were related to transcriptional activity [36]. Despite this, we were able to identify several markers that are involved in metabolism and/or the actions of steroid hormones (Table 6). The HVC enrichment of the corticotropin-releasing hormone binding protein (CRHBP; Fig. 2B) and of a mineralocorticoid receptor (NR3C2) suggests as yet undefined roles of corticoid-related mechanisms in modulating HVC properties, possibly in a very distinct and sparse subset of cells.

Sex steroids (i.e. androgens and estrogens) are known to play prominent roles in sexual dimorphism, the development and physiology of the song system, and adult neurogenesis. Interestingly, despite the pervasive effects of androgens in HVC, we identified only 3 genes with known androgen-related action. We found a similarly small number of genes, including CYP19A1 (i.e. aromatase), that are known to participate in the metabolism or to mediate the actions of estrogens. This might be due to the relatively small number of androgen and/or estrogen-related targets characterized to date. In light of the known distribution of androgen and estrogen receptors (predominantly in HVC and Shelf, respectively), we suggest that the identified androgen- and estrogen-related markers result from differential gene regulation in the HVC and Shelf respectively, consistent with the notion that these areas may be predominantly under androgenic and estrogenic regulation, respectively. Future analyses of genome binding sites for estrogen and androgen receptors may provide a more comprehensive method for identifying additional steroid target genes in the song system.

The Vitamin A metabolite retinoic acid has broad actions during development and cellular differentiation. In songbirds, it is required for the maturation of song and is specifically synthesized in HVC's X-projecting neurons by the enzyme zRalDH [24]. Our analysis has revealed that a broad range of HVC markers have been linked to retinoic acid metabolism and/or action in other systems, and thus constitute potential modulators or effectors of the actions of retinoic acid on song behavior. In particular, several specific signaling pathways (wnt, notch, BMP, MYCN, cell adhesion pathways) were identified that may help future studies to establish the mechanisms of retinoic acid action on the song system and singing behavior. Interestingly, the overall balance of expression of retinoid-related HVC markers suggests a general suppression of growth- and development-related pathways and an enhancement of stabilizing cell adhesion specializations and neuronal differentiation.

### Conclusions

Overall, our results provide the most comprehensive characterization to date of molecular genetic specializations of HVC that are likely related to the unique properties of this song nucleus. Specifically, our results add: (1) more than 200 novel HVC markers, including genes that may be markers of specific cell populations, (2) information about likely targets for pharmacological and/or genetic manipulations, thus providing a wealth of possible new tools that songbird researchers could use to dissect the physiology of the song system, and (3) new perspectives on the roles that individual genes and genetic pathways might play in various aspects of cellular morphogenesis, excitability, neurotransmission, neurogenesis and cell survival, steroid and retinoid metabolism and sensitivity, and gene regulation in HVC. Future studies will be directed at determining whether and/or how these genes may modulate different aspects of song production and/or learning, the main functions of song nucleus HVC.

## Materials and Methods

### Animals

All birds were adult male zebra finches (>120 days, *Taeniopygia gutatta*) from our breeding colony or local breeders. To minimize the induction of activity-regulated genes by singing behavior and auditory stimulation [55], [56], and thus maximize the identification of bona fide molecular specializations of HVC, whose expression is not dependent on the state of neuronal activation, all birds were isolated overnight (16–20 hr) in sound-attenuated chambers under a 12∶12 light∶dark cycle and monitored to ensure lack of singing for at least 2 hr prior to being sacrificed by decapitation between 9 and 11 AM. We specifically used this protocol because it largely resembles what occurs naturally during the night period (i.e. minimal vocal-motor behavior or auditory stimulation). It also corresponds to a basal control condition that we, and others (e.g. [57]–[59]) have used to demonstrate the regulated expression of activity-dependent genes in the contexts of hearing and singing, while minimizing the duration of social separation. The brains were quickly dissected, frozen in a dry ice-isopropanol bath, sectioned parasagittally on a cryostat, thaw-mounted on pre-cleaned slides (2 left-hemisphere 10 µm sections per slide; Thermo Fisher Scientific, Waltham, MA), and stored at -80°C. All procedures conformed to NIH guidelines for the use and care of animals in research and were approved by OHSU's Institutional Animal Care Use Committee (IACUC).

### Sample preparation

For each bird (n = 6) a set of 10 slides was selected that contained sections through HVC (∼1.4 to 2.4 mm from the midline) and the immediately adjacent nidopallial Shelf area (Fig. 1A). Tissue preparation followed established protocols (Arcturus, Mountainview, CA), and all solutions were made using autoclaved DEPC-H_2_O (DH_2_O), fresh alcohols, and xylenes to minimize RNAse contamination. Briefly, slides were removed from the −80°C freezer, rinsed briefly in DH_2_O, fixed in 70% ethanol (30 sec), washed in DH_2_O (15–30 sec), stained with 1% cresyl violet in 1 M sodium acetate (∼30 sec), rinsed 2× with DH_2_O, dehydrated through a graded ethanol series (70%, 95%, 100%, 100%, 30 sec each), and transferred to xylenes for at least 5 min. Slides were air dried for 5 min and transferred to an RNAse-free slide box containing desiccant.

HVC was identified under bright-field illumination by the characteristic bump on the brain surface and the presence of clusters of large- to medium-sized ovoid-shaped cells oriented parallel to the overlying ventricle (Fig. 1B), or under dark-field by the contrasting density of myelinated fibers close to the ventral boundary of HVC and the fiber bundles exiting caudal HVC (Fig. 1C). HVC dissections were performed conservatively with respect to its boundaries (Fig. 1D) and confirmed following dissections (Fig. 1E), ensuring that the overlying hippocampus was not included. For the Shelf we sampled a 300 µm thick region at the same rostral-caudal level but ∼100 µm ventral to HVC, thus well within the Shelf as defined by connectivity (see Figs. 12 and 13 in [60]).

We captured HVC or Shelf samples (n = 20 sections) onto CapSure Macro caps on an Arcturus PixCellII system (Mountainview, CA) mounted on a Nikon microscope at 10–20× using standard laser settings. Tissue not embedded in the cap was removed by application of CapSure strips and confirmation of captures was made by visual inspection. Caps were then placed in an Extractsure device (Arcturus), 10 µl of lysis buffer with β-mercaptoethanol (0.7 µl per 100 µl lysis buffer; Nanoprep, Stratagene) was added, and the resulting lysate retrieved. After adding 40 µl of lysis buffer, each sample was vortexed (1 min), triturated by repeated pipetting, and stored on ice. RNA was extracted using Stratagene's Absolutely RNA Nanoprep kit and assessed by spectrophotometry (ND-1000; Nanodrop). For each bird, the HVC or Shelf samples from all selected sections were pooled for further analysis.

### Microarray analysis

RNA sample amplification, dye labeling, and microarray hybridization was conducted as part of Community Collaboration under the Songbird Neurogenomics Initiative and used a universal reference design [23]. Spotted cDNA glass microarrays encompassed 19,213 individual cDNAs corresponding to 17,214 unique genes (1,900 possibly representing splice variants), 1,631 replicate clones, and 368 control genes and knowns ([23]; “20 k” arrays, SB03 build of the ESTIMA:Songbird collection; http://titan.biotec.uiuc.edu/cgi-bin/ESTWebsite/estima_startseqSetsongbird). Based on sequence similarity searches against the International Protein Index (European Bioinformatics Institute; [61]), 7,898 (44%) ESTs have annotations.

RNA samples were subjected to two rounds of linear amplification (MessageAmpII kit; Ambion). The resulting aRNAs were reverse transcribed using indirect aminoallyl incorporation and labeled with either Cy3 or Cy5. Using a universal design, dye labeling was balanced by group and each aRNA (from HVC or Shelf) was hybridized against a common reference RNA (pooled and amplified RNA from whole telencephalon of 15 male and 15 female adult zebra finches; [23]). Thus each array represents a biological replicate measure of RNA from an HVC or Shelf sample compared to the reference.

Each probe pair (HVC or Shelf vs. reference) was hybridized to the arrays overnight at 42°C, washed and scanned using an Axon GenePix 4000B microarray scanner, and analyzed using GenePix Pro 6.0 software to flag aberrant spots (as in [23]). Using GeneSpring (GE Healthcare) and ARDAS 2.0 (Array Repository and Data Analysis System; 3rd Millennium), datasets for HVC and Shelf samples were Log_2_-transformed and loess normalized within each array. Normalized data were then adjusted for both global array and dye effects, converted into fold-ratios of signal intensity for each sample versus reference, and compared across samples (HVC vs. Shelf) using conservative one-way ANOVA with Benjamini and Hochberg post hoc t-tests (GeneSpring) or a parametric linear mixed effects model implemented in bioconductor R (www.bioconductor.org; after [62], [63]). Our primary list of candidate HVC markers (Table S1) was generated first in GeneSpring (FDR<0.05), and then cross-referenced as a confirmatory step to a list derived from ARDAS (FDR<0.01). We also generated a secondary candidate list (Table S2) consisting of genes that were differential in GeneSpring (FDR<0.1) or had the same pattern of regulation (either up or down) in 5/6 out of 6 sample pairs, and whose function was related to genes on our primary list. All of the raw microarray data are available in a MIAME-compliant format, and can be accessed at the following URL: <http://128.174.53.227:8080/ardas/install.html>, using “public” (no quotes) as the login and password.

### Bioinformatics Analysis

Only ∼37% of the ESTs that showed differential expression at the FDR<0.05 level had ESTIMA (songbird3) annotations that allowed for further inquiry into gene function. To confirm annotations and identify unannotated clones, we used EST alignments against the chicken genome via blat (http://genome.ucsc.edu/), and megablast searches of GenBank databases. In most cases we were able to either confirm or establish an identification based on the EST alignment to several annotated transcriptional units (TU; i.e. RefSeqs or mRNAs) that also mapped onto the chicken genome. In cases where a zebra finch EST mapped within a few hundred (∼500) base-pairs of a given TU, positive identification was only made if a connection between the zebra finch EST and that TU could be established through overlapping chicken ESTs. Throughout the results, we use the HUGO system for gene names and abbreviations to indicate homology with human genes (http://www.genenames.org/); tentative identifications are underlined in the text and tables.

To facilitate the discovery of specific biological processes or molecular functions that might be targets of regulation in HVC, we used AgBase (http://www.agbase.msstate.edu/) to assign gene ontology (GO) terms to as many genes as possible from our FDR<0.05 list, and then compared these terms to a set obtained for 1415 ESTIMA unigenes representing a broad sample of the possible universe of genes expressed in the songbird brain. Thus, this analysis compared a set of GO terms derived from the set of genes found to be differential between HVC and Shelf with a second set of terms that more generally describe the properties of the brain as whole. We then plotted the percentage of genes in either the FDR<0.05 list or the broader ESTIMA that fell into the 241 molecular function, 263 biological process, and 88 cell compartmentalization categories identified. From these, we selected the subset that minimally described 3% of the genes in the FDR<0.05 list (i.e., contained at least 10–12 genes), and then identified categories that were under- or over-represented (i.e. had a higher or lower percentage of hits in the FDR<0.05 list than in the broader list from ESTIMA).

To identify genetic and/or biochemical pathways that might be targets of differential regulation in HVC vs. Shelf, we performed extensive database and literature searches. For some markers, groupings according to cellular/biochemical function could be easily derived from their identities (e.g., ion channels, receptors, etc). For others, Entrez Gene (www.ncbi.nlm.nih.gov/entrez/query.fcgidbgene) provided highly useful summaries, GO terms, links to specific Kegg pathways, and relevant literature compilations to establish functions and relationships. For a small number of genes we recurred to PubMed searches on the recent (last 5 years) literature with regards to specific function and/or expression in brain tissue (presented in References S1). Using this combined approach, we were able to categorize about 82% of the annotated markers in our FDR<0.05 according to a subset of descriptive cellular functions and pathways (Tables 2–7).

### *In situ* hybridization

^33^P-labelled sense and antisense riboprobes were hybridized to serial parasagittal sections of additional (n = 4–6) adult male zebra finches followed by phosphorimager autoradiography (Typhoon 9410, GE Healthcare) for a global assessment of brain mRNA distribution, or emulsion autoradiography followed by Nissl (cresyl violet or toluidine blue) counterstaining for regional and cellular analysis. Hybridizations and washes were performed at 65°C, as previously described [64]. In some cases marker expression was mapped using Neurolucida software integrated with a Nikon E-600 microscope with a motorized stage drive and coupled to a PC through a Lucivid system (Microbrightfield; Colchester, VT). To confirm the differential expression of HVC candidate markers, we used ImageJ software (NIH) to quantify levels of expression in HVC and Shelf based on optical density (OD) following Gel-Data Linearization (http://rsb.info.nih.gov/ij/plugins/linearize-gel-data.html). For each pair of HVC and Shelf measurements, we also measured and subtracted signal intensity measured over the glass background in an area immediately adjacent to each section to account for any non-specific stickiness of probe to glass, and control for possible image artifacts (e.g. unevenness in phosphorimager screen). ANOVA's were used to test for group differences in expression levels for each candidate gene.

## Supporting Information

References S1 (0.09 MB DOC)

Table S1 Primary FDR<0.05 HVC Markers.
(0.08 MB PDF)

Table S2 Secondary List of Differential Expressed HVC Markers.
(0.06 MB PDF)

Table S3 Previously Confirmed Markers of HVC.
(0.04 MB PDF)

Table S4 Microarray validations by in situ hybridization
(0.08 MB PDF)

Table S5 Extended search Genelist (ND).
(0.06 MB PDF)
