# Supplementary material for: Birdsong “Transcriptomics”: Neurochemical Specializations of the Oscine Song System
Source: PLoS One. 2008 Oct 20;3(10):e3440. doi: 10.1371/journal.pone.0003440 (PMC2563692; doi:10.1371/journal.pone.0003440)
Supplement: References S1 — (0.09 MB DOC) [file pone.0003440.s001.doc]

**References S1**

1. Adachi K, Toyota M, Sasaki Y, Yamashita T, Ishida S, Ohe-Toyota M, Maruyama R, Hinoda Y, Saito T, Imai K, Kudo R, Tokino T. Identification of SCN3B as a novel p53-inducible proapoptotic gene. Oncogene. 2004 Oct 14;23(47):7791-8.

2. Afink GB, Nistér M, Stassen BH, Joosten PH, Rademakers PJ, Bongcam-Rudloff E, Van Zoelen EJ, Mosselman S. Molecular cloning and functional characterization of the human platelet-derived growth factor alpha receptor gene promoter. Oncogene. 1995 Apr 20;10(8):1667-72

3. Agate RJ, Hertel M, Nottebohm F. FnTm2, a novel brain-specific transcript, is dynamically expressed in the song learning circuit of the zebra finch. J Comp Neurol. 2007 Sep 10;504(2):127-4

4. Angata K, Suzuki M, Fukuda M. ST8Sia II and ST8Sia IV polysialyltransferases exhibit marked differences in utilizing various acceptors containing oligosialic acid and short polysialic acid. The basis for cooperative polysialylation by two enzymes. J Biol Chem. 2002 Sep 27;277(39):36808-17.

5. Arima, Kayo, Jason Shiotsugu, Rong Niu, Ritika Khandpur, Mayra Martinez, Yongchol Shin, Tetsuya Koide, Ken W. Y. Cho, Atsushi Kitayama, Naoto Ueno, Roshantha A. S. Chandraratna, and Bruce Blumberg. Global analysis of RAR-responsive genes in the Xenopus neurula using cDNA microarrays. Dev. Dynam. 232, no. 2: 414-31.

6. Balcerzak M, Bandorowicz-Pikula J, Buchet R, Pikula S. A novel retinoid binding property of human annexin A6. FEBS Lett. 2006 May 29;580(13):3065-9.

7. Baldassa S, Gnesutta N, Fascio U, Sturani E, Zippel R. SCLIP, a microtubule-destabilizing factor, interacts with RasGRF1 and inhibits its ability to promote Rac activation and neurite outgrowth. J Biol Chem. 2007 Jan 26;282(4):2333-45.

8. Becker W, Heukelbach J, Kentrup H, Joost HG. Molecular cloning and characterization of a novel mammalian protein kinase harboring a homology domain that defines a subfamily of serine/threonine kinases. Eur J Biochem. 1996 Feb 1;235(3):736-43.

9. Berezowski V, Landry C, Dehouck MP, Cecchelli R, Fenart L. Contribution of glial cells and pericytes to the mRNA profiles of P-glycoprotein and multidrug resistance-associated proteins in an in vitro model of the blood-brain barrier. Brain Res. 2004 Aug 20;1018(1):1-9.

10. Bilezikjian LM, Blount AL, Donaldson CJ, Vale WW. Pituitary actions of ligands of the TGF-beta family: activins and inhibins. Reproduction. 2006 Aug;132(2):207-15. Review.

11. Bohl J, Brimer N, Lyons C, Vande Pol SB. The stardust family protein MPP7 forms a tripartite complex with LIN7 and DLG1 that regulates the stability and localization of DLG1 to cell junctions. J Biol Chem. 2007 Mar 30;282(13):9392-400.

12. Brenneke F, Schachner M, Elger CE, Lie AA. Up-regulation of the extracellular matrix glycoprotein tenascin-R during axonal reorganization and astrogliosis in the adult rat hippocampus. Epilepsy Res. 2004 Feb;58(2-3):133-43

13. Bu X, Avraham HK, Li X, Lim B, Jiang S, Fu Y, Pestell RG, Avraham S. Mayven induces c-Jun expression and cyclin D1 activation in breast cancer cells. Oncogene. 2005 Mar 31;24(14):2398-409.

14. Calabrese B, Halpain S. Essential role for the PKC target MARCKS in maintaining dendritic spine morphology. Neuron. 2005 Oct 6;48(1):77-90.

15. Chao CC, Chang PY, Lu HH. Human Gas7 isoforms homologous to mouse transcripts differentially induce neurite outgrowth. J Neurosci Res. 2005 Jul 15;81(2):153-62

16. Chauhan S, Pandey R, Way JF, Sroka TC, Demetriou MC, Kunz S, Cress AE, Mount DW, Miesfeld RL. Androgen regulation of the human FERM domain encoding gene EHM2 in a cell model of steroid-induced differentiation. Biochem Biophys Res Commun. 2003 Oct 17;310(2):421-32.

17. Cook KK, Fadool DA. Two adaptor proteins differentially modulate the phosphorylation and biophysics of Kv1.3 ion channel by SRC kinase. J Biol Chem. 2002 Apr 12;277(15):13268-80.

18. Dan I, Ong SE, Watanabe NM, Blagoev B, Nielsen MM, Kajikawa E, Kristiansen TZ, Mann M, Pandey A. Cloning of MASK, a novel member of the mammalian germinal center kinase III subfamily, with apoptosis-inducing properties. J Biol Chem. 2002 Feb 22;277(8):5929-39.

19. Ebinger M, Senf L, Wachowski O, Scheurlen W. Expression of GAS7 in childhood CNS tumors. Pediatr Blood Cancer. 2006 Mar;46(3):325-8.

20. Englund C, Fink A, Lau C, Pham D, Daza RA, Bulfone A, Kowalczyk T, Hevner RF. Pax6, Tbr2, and Tbr1 are expressed sequentially by radial glia, intermediate progenitor cells, and postmitotic neurons in developing neocortex. J Neurosci. 2005 Jan 5;25(1):247-51.

21. Freemantle SJ, Kerley JS, Olsen SL, Gross RH, Spinella MJ. Developmentally-related candidate retinoic acid target genes regulated early during neuronal differentiation of human embryonal carcinoma. Oncogene. 2002 Apr 25;21(18):2880-9.

22. Gagliardi AD, Kuo EY, Raulic S, Wagner GF, DiMattia GE. Human stanniocalcin-2 exhibits potent growth-suppressive properties in transgenic mice independently of growth hormone and IGFs. Am J Physiol Endocrinol Metab. 2005 Jan;288(1):E92-105.

23. Gabrielli F, Donadel G, Bensi G, Heguy A, Melli M. A nuclear protein, synthesized in growth-arrested human hepatoblastoma cells, is a novel member of the short-chain alcohol dehydrogenase family. Eur J Biochem. 1995 Sep 1;232(2):473-7.

24. Gentil BJ, Benaud C, Delphin C, Remy C, Berezowski V, Cecchelli R, Feraud O, Vittet D, Baudier J. Specific AHNAK expression in brain endothelial cells with barrier properties. J Cell Physiol. 2005 May;203(2):362-71.

25. Gollogly LK, Ryeom SW, Yoon SS. Down syndrome candidate region 1-like 1 (DSCR1-L1) mimics the inhibitory effects of DSCR1 on calcineurin signaling in endothelial cells and inhibits angiogenesis. J Surg Res. 2007 Sep;142(1):129-36.

26. Gu Z, Jiang Q, Yan Z. RGS4 modulates serotonin signaling in prefrontal cortex and links to serotonin dysfunction in a rat model of schizophrenia. Mol Pharmacol. 2007 Apr;71(4):1030-9. Epub 2007 Jan 12.

27. Hassel S, Eichner A, Yakymovych M, Hellman U, Knaus P, Souchelnytskyi S. Proteins associated with type II bone morphogenetic protein receptor (BMPR-II) and identified by two-dimensional gel electrophoresis and mass spectrometry. Proteomics. 2004 May;4(5):1346-58.

28. Hattori H, Zhang X, Jia Y, Subramanian KK, Jo H, Loison F, Newburger PE, Luo HR. RNAi screen identifies UBE2D3 as a mediator of all-trans retinoic acid-induced cell growth arrest in human acute promyelocytic NB4 cells. Blood. 2007 Jul 15;110(2):640-50.

29. Hiramoto K, Negishi M, Katoh H. Dock4 is regulated by RhoG and promotes Rac-dependent cell migration. Exp Cell Res. 2006 Dec 10;312(20):4205-16.

30. Hirrlinger J, König J, Dringen R. Expression of mRNAs of multidrug resistance proteins (Mrps) in cultured rat astrocytes, oligodendrocytes, microglial cells and neurones. J Neurochem. 2002 Aug;82(3):716-9.

31. Hirsch E, Pozzato M, Vercelli A, Barberis L, Azzolino O, Russo C, Vanni C, Silengo L, Eva A, Altruda F. Defective dendrite elongation but normal fertility in mice lacking the Rho-like GTPase activator Dbl. Mol Cell Biol. 2002 May;22(9):3140-8.

32. Hollway GE, Maule J, Gautier P, Evans TM, Keenan DG, Lohs C, Fischer D, Wicking C, Currie PD. Scube2 mediates Hedgehog signalling in the zebrafish embryo. Dev Biol. 2006 Jun 1;294(1):104-18.

33. Hongo S, Watanabe T, Takahashi K, Miyazaki A. Ndrg4 enhances NGF-induced ERK activation uncoupled with Elk-1 activation. J Cell Biochem. 2006 May 1;98(1):185-93.

34. Huentelman MJ, Papassotiropoulos A, Craig DW, Hoerndli FJ, Pearson JV, Huynh KD, Corneveaux J, Hänggi J, Mondadori CR, Buchmann A, Reiman EM, Henke K, de Quervain DJ, Stephan DA. Calmodulin-binding transcription activator 1 (CAMTA1) alleles predispose human episodic memory performance. Hum Mol Genet. 2007 Jun 15;16(12):1469-77.

35. Hussain RJ, Stumpo DJ, Blackshear PJ, Lenox RH, Abel T, McNamara RK. Myristoylated alanine rich C kinase substrate (MARCKS) heterozygous mutant mice exhibit deficits in hippocampal mossy fiber-CA3 long-term potentiation. Hippocampus. 2006;16(5):495-503.

36. Ishisaki Z, Takaishi M, Furuta I, Huh N. Calmin, a protein with calponin homology and transmembrane domains expressed in maturing spermatogenic cells.Genomics. 2001 Jun 1;74(2):172-9.

37. Jerng HH, Lauver AD, Pfaffinger PJ. DPP10 splice variants are localized in distinct neuronal populations and act to differentially regulate the inactivation properties of Kv4-based ion channels. Mol Cell Neurosci. 2007 Aug;35(4):604-24.

38. Jiang S, Avraham HK, Park SY, Kim TA, Bu X, Seng S, Avraham S. Process elongation of oligodendrocytes is promoted by the Kelch-related actin-binding protein Mayven. J Neurochem. 2005 Mar;92(5):1191-203.

39. Jiang Y, Liu YE, Lu A, Gupta A, Goldberg ID, Liu J, Shi YE. Stimulation of estrogen receptor signaling by gamma synuclein. Cancer Res. 2003 Jul 15;63(14):3899-903.

40. Kanemaru KK, Tuthill MC, Takeuchi KK, Sidell N, Wada RK. Retinoic acid induced downregulation of MYCN is not mediated through changes in Sp1/Sp3. Pediatr Blood Cancer. 2008 Apr;50(4):806-11.

41. Kang SW, Shin YJ, Shim YJ, Jeong SY, Park IS, Min BH. Clusterin interacts with SCLIP (SCG10-like protein) and promotes neurite outgrowth of PC12 cells. Exp Cell Res. 2005 Oct 1;309(2):305-15.

42. Kikuchi R, Sobue S, Murakami M, Ito H, Kimura A, Iwasaki T, Shibayama S, Takagi A, Kojima T, Suzuki M, Banno Y, Nozawa Y, Murate T. Mechanism of vitamin D3-induced transcription of phospholipase D1 in HaCat human keratinocytes. FEBS Lett. 2007 May 1;581(9):1800-4.

43. Klüppel M, Vallis KA, Wrana JL. A high-throughput induction gene trap approach defines C4ST as a target of BMP signaling. Mech Dev. 2002 Oct;118(1-2):77-89.

44. Ko JA, Kimura Y, Matsuura K, Yamamoto H, Gondo T, Inui M. PDZRN3 (LNX3, SEMCAP3) is required for the differentiation of C2C12 myoblasts into myotubes. J Cell Sci. 2006 Dec 15;119(Pt 24):5106-13.

45. Kobayashi T, Masaki T, Sugiyama M, Atomi Y, Furukawa Y, Nakamura Y. A gene encoding a family with sequence similarity 84, member A (FAM84A) enhanced migration of human colon cancer cells. Int J Oncol. 2006 Aug;29(2):341-7

46. Komai K, Mukae-Sakairi N, Kitagawa M, Shiozawa S. Characterization of novel splicing variants of the mouse MCF-2 (DBL) proto-oncogene. Biochem Biophys Res Commun. 2003 Oct 3;309(4):906-9.

47. Korkeamäki H, Viiri K, Kukkonen MK, Mäki M, Lohi O. Alternative mRNA splicing of SAP30L regulates its transcriptional repression activity. FEBS Lett. 2008 Jan 23;582(2):379-84.

48. Krummenacher C, Baribaud F, Ponce de Leon M, Baribaud I, Whitbeck JC, Xu R, Cohen GH, Eisenberg RJ.Comparative usage of herpesvirus entry mediator A and nectin-1 by laboratory strains and clinical isolates of herpes simplex virus. Virology. 2004 May 1;322(2):286-99.

49. Ku M, Howard S, Ni W, Lagna G, Hata A. OAZ regulates bone morphogenetic protein signaling through Smad6 activation. J Biol Chem. 2006 Feb 24;281(8):5277-87.

50. Kuang WW, Thompson DA, Hoch RV, Weigel RJ. Differential screening and suppression subtractive hybridization identified genes differentially expressed in an estrogen receptor-positive breast carcinoma cell line. Nucleic Acids Res. 1998 Feb 15;26(4):1116-23.

51. Kumar S, Sieghart W, Morrow AL. Association of protein kinase C with GABA(A) receptors containing alpha1 and alpha4 subunits in the cerebral cortex: selective effects of chronic ethanol consumption. J Neurochem. 2002 Jul;82(1):110-7.

52. Kwon KB, Park EK, Ryu DG, Park BH. D4-GDI is cleaved by caspase-3 during daunorubicin-induced apoptosis in HL-60 cells. Exp Mol Med. 2002 Mar 31;34(1):32-7.

53. Lansbergen G, Grigoriev I, Mimori-Kiyosue Y, Ohtsuka T, Higa S, Kitajima I, Demmers J, Galjart N, Houtsmuller AB, Grosveld F, Akhmanova A. CLASPs attach microtubule plus ends to the cell cortex through a complex with LL5beta. Dev Cell. 2006 Jul;11(1):21-32.

54. Laube G, Seidenbecher CI, Richter K, Dieterich DC, Hoffmann B, Landwehr M, Smalla KH, Winter C, Böckers TM, Wolf G, Gundelfinger ED, Kreutz MR. The neuron-specific Ca2+-binding protein caldendrin: gene structure, splice isoforms, and expression in the rat central nervous system. Mol Cell Neurosci. 2002 Mar;19(3):459-75.

55. Lee-Hoeflich ST, Causing CG, Podkowa M, Zhao X, Wrana JL, Attisano L. Activation of LIMK1 by binding to the BMP receptor, BMPRII, regulates BMP-dependent dendritogenesis. EMBO J. 2004 Dec 8;23(24):4792-801.

56. Li J, O'Connor KL, Greeley GH Jr, Blackshear PJ, Townsend CM Jr, Evers BM. Myristoylated alanine-rich C kinase substrate-mediated neurotensin release via protein kinase C-delta downstream of the Rho/ROK pathway. J Biol Chem. 2005 Mar 4;280(9):8351-7.

57. Li Y, Suino K, Daugherty J, Xu HE. Structural and biochemical mechanisms for the specificity of hormone binding and coactivator assembly by mineralocorticoid receptor. Mol Cell. 2005 Aug 5;19(3):367-80

58. Liao H, Bu WY, Wang TH, Ahmed S, Xiao ZC. Tenascin-R plays a role in neuroprotection via its distinct domains that coordinate to modulate the microglia function. J Biol Chem. 2005 Mar 4;280(9):8316-23.

59. Lin KR, Lee SF, Hung CM, Li CL, Yang-Yen HF, Yen JJ. Survival factor withdrawal-induced apoptosis of TF-1 cells involves a TRB2-Mcl-1 axis-dependent pathway. J Biol Chem. 2007 Jul 27;282(30):21962-72.

60. Lindfors K, Viiri KM, Niittynen M, Heinonen TY, Mäki M, Kainulainen H. TGF-beta induces the expression of SAP30L, a novel nuclear protein. BMC Genomics. 2003 Dec 18;4(1):53.

61. Lortie K, Huang D, Chakravarthy B, Comas T, Hou ST, Lin-Chao S, Morley P. The gas7 protein potentiates NGF-mediated differentiation of PC12 cells. Brain Res. 2005 Mar 2;1036(1-2):27-34.

62. Lu Z, Je HS, Young P, Gross J, Lu B, Feng G. Regulation of synaptic growth and maturation by a synapse-associated E3 ubiquitin ligase at the neuromuscular junction. J Cell Biol. 2007 Jun 18;177(6):1077-89.

63. Lui WY, Lee WM, Cheng CY. Sertoli-germ cell adherens junction dynamics in the testis are regulated by RhoB GTPase via the ROCK/LIMK signaling pathway. Biol Reprod. 2003 Jun;68(6):2189-206.

64. Luxardi G, Galli A, Forlani S, Lawson K, Maina F, Dono R. Glypicans are differentially expressed during patterning and neurogenesis of early mouse brain. Biochem Biophys Res Commun. 2007 Jan 5;352(1):55-60. Epub 2006 Nov 10.

65. Lyman SK, Gerace L, Baserga SJ. Human Nop5/Nop58 is a component common to the box C/D small nucleolar ribonucleoproteins. RNA. 1999 Dec;5(12):1597-604.

66. Ma X, Zhao H, Shan J, Long F, Chen Y, Chen Y, Zhang Y, Han X, Ma D. PDCD10 interacts with Ste20-related kinase MST4 to promote cell growth and transformation via modulation of the ERK pathway. Mol Biol Cell. 2007 Jun;18(6):1965-78.

67. Martinez-Ceballos E, Chambon P, Gudas LJ. Differences in gene expression between wild type and Hoxa1 knockout embryonic stem cells after retinoic acid treatment or leukemia inhibitory factor (LIF) removal. J Biol Chem. 2005 Apr 22;280(16):16484-98.

68. McFarland KN, Wilkes SR, Koss SE, Ravichandran KS, Mandell JW. Neural-specific inactivation of ShcA results in increased embryonic neural progenitor apoptosis and microencephaly. J Neurosci. 2006 Jul 26;26(30):7885-97.

69. Merrill RA, Ahrens JM, Kaiser ME, Federhart KS, Poon VY, Clagett-Dame M. All-trans retinoic acid-responsive genes identified in the human SH-SY5Y neuroblastoma cell line and their regulated expression in the nervous system of early embryos. Biol Chem. 2004 Jul;385(7):605-14.

70. Nadal MS, Amarillo Y, Vega-Saenz de Miera E, Rudy B. Differential characterization of three alternative spliced isoforms of DPPX. Brain Res. 2006 Jun 13;1094(1):1-12.

71. Nelson SA, Santora KE, LaRochelle WJ. Isolation and characterization of a novel PDGF-induced human gene. Gene. 2000 Jul 25;253(1):87-93.

72. Niu S, Renfro A, Quattrocchi CC, Sheldon M, D'Arcangelo G. Reelin promotes hippocampal dendrite development through the VLDLR/ApoER2-Dab1 pathway. Neuron. 2004 Jan 8;41(1):71-84.

73. O'Kane EM, Stone TW, Morris BJ. Activation of Rho GTPases by synaptic transmission in the hippocampus. J Neurochem. 2003 Dec;87(5):1309-12.

74. Oh DY, Park SY, Cho JH, Lee KS, Min do S, Han JS. Phospholipase D1 activation through Src and Ras is involved in basic fibroblast growth factor-induced neurite outgrowth of H19-7 cells. J Cell Biochem. 2007 May 1;101(1):221-34.

75. Ohki T, Hongo S, Nakada N, Maeda A, Takeda M. Inhibition of neurite outgrowth by reduced level of NDRG4 protein in antisense transfected PC12 cells. Brain Res Dev Brain Res. 2002 Apr 30;135(1-2):55-63.

76. Perillan PR, Chen M, Potts EA, Simard JM. Transforming growth factor-beta 1 regulates Kir2.3 inward rectifier K+ channels via phospholipase C and protein kinase C-delta in reactive astrocytes from adult rat brain. J Biol Chem. 2002 Jan 18;277(3):1974-80.

77. Postigo AA. Opposing functions of ZEB proteins in the regulation of the TGFbeta/BMP signaling pathway. EMBO J. 2003 May 15;22(10):2443-5

78. Postigo AA, Depp JL, Taylor JJ, Kroll KL. Regulation of Smad signaling through a differential recruitment of coactivators and corepressors by ZEB proteins. EMBO J. 2003 May 15;22(10):2453-62.

79. Qin P, Cimildoro R, Kochhar DM, Soprano KJ, Soprano DR. PBX, MEIS, and IGF-I are potential mediators of retinoic acid-induced proximodistal limb reduction defects. Teratology. 2002 Nov;66(5):224-34.

80. Qiu J, Bosch MA, Jamali K, Xue C, Kelly MJ, Rønnekleiv OK. Estrogen upregulates T-type calcium channels in the hypothalamus and pituitary. J Neurosci. 2006 Oct 25;26(43):11072-82.

81. Qiu J, Bosch MA, Tobias SC, Grandy DK, Scanlan TS, Ronnekleiv OK, Kelly MJ. Rapid signaling of estrogen in hypothalamic neurons involves a novel G-protein-coupled estrogen receptor that activates protein kinase C. J Neurosci. 2003 Oct 22;23(29):9529-40.

82. Richman RW, Strock J, Hains MD, Cabanilla NJ, Lau KK, Siderovski DP, Diversé-Pierluissi M. RGS12 interacts with the SNARE-binding region of the Cav2.2 calcium channel. J Biol Chem. 2005 Jan 14;280(2):1521-8.

83. Rimler A, Jockers R, Lupowitz Z, Zisapel N. Gi and RGS proteins provide biochemical control of androgen receptor nuclear exclusion. J Mol Neurosci. 2007;31(1):1-12.

84. Saghatelyan A, de Chevigny A, Schachner M, Lledo PM. Tenascin-R mediates activity-dependent recruitment of neuroblasts in the adult mouse forebrain. Nat Neurosci. 2004 Apr;7(4):347-56.

85. Sambi BS, Hains MD, Waters CM, Connell MC, Willard FS, Kimple AJ, Pyne S, Siderovski DP, Pyne NJ. The effect of RGS12 on PDGFbeta receptor signalling to p42/p44 mitogen activated protein kinase in mammalian cells.Cell Signal. 2006 Jul;18(7):971-81.

86. Schaapveld RQ, van den Maagdenberg AM, Schepens JT, Weghuis DO, Geurts van Kessel A, Wieringa B, Hendriks WJ. The mouse gene Ptprf encoding the leukocyte common antigen-related molecule LAR: cloning, characterization, and chromosomal localization. Genomics. 1995 May 1;27(1):124-30.

87. Schunke D, Span P, Ronneburg H, Dittmer A, Vetter M, Holzhausen HJ, Kantelhardt E, Krenkel S, Müller V, Sweep FC, Thomssen C, Dittmer J. Cyclooxygenase-2 is a target gene of rho GDP dissociation inhibitor beta in breast cancer cells. Cancer Res. 2007 Nov 15;67(22):10694-702.

88. Seo S, Richardson GA, Kroll KL. The SWI/SNF chromatin remodeling protein Brg1 is required for vertebrate neurogenesis and mediates transactivation of Ngn and NeuroD. Development. 2005 Jan;132(1):105-15.

89. Serra-Pagès C, Medley QG, Tang M, Hart A, Streuli M. Liprins, a family of LAR transmembrane protein-tyrosine phosphatase-interacting proteins. J Biol Chem. 1998 Jun 19;273(25):15611-20.

90. Shirahata E, Iwasaki H, Takagi M, Lin C, Bennett V, Okamura Y, Hayasaka K. Ankyrin-G regulates inactivation gating of the neuronal sodium channel, Nav1.6. J Neurophysiol. 2006 Sep;96(3):1347-57.

91. Shorts-Cary L, Xu M, Ertel J, Kleinschmidt-Demasters BK, Lillehei K, Matsuoka I, Nielsen-Preiss S, Wierman ME. Bone morphogenetic protein and retinoic acid-inducible neural specific protein-3 is expressed in gonadotrope cell pituitary adenomas and induces proliferation, migration, and invasion. Endocrinology. 2007 Mar;148(3):967-75.

92. Singhrao SK, Neal JW, Rushmere NK, Morgan BP, Gasque P. Differential expression of individual complement regulators in the brain and choroid plexus.Lab Invest. 1999 Oct;79(10):1247-59.

93. Speidel D, Varoqueaux F, Enk C, Nojiri M, Grishanin RN, Martin TF, Hofmann K, Brose N, Reim K. A family of Ca2+-dependent activator proteins for secretion: comparative analysis of structure, expression, localization, and function. J Biol Chem. 2003 Dec 26;278(52):52802-9. Epub 2003 Oct 6.

94. Suto F, Ito K, Uemura M, Shimizu M, Shinkawa Y, Sanbo M, Shinoda T, Tsuboi M, Takashima S, Yagi T, Fujisawa H. Plexin-a4 mediates axon-repulsive activities of both secreted and transmembrane semaphorins and plays roles in nerve fiber guidance. J Neurosci. 2005 Apr 6;25(14):3628-37.

95. Takaishi M, Ishisaki Z, Yoshida T, Takata Y, Huh NH. Expression of calmin, a novel developmentally regulated brain protein with calponin-homology domains. Brain Res Mol Brain Res. 2003 Apr 10;112(1-2):146-52.

96. Trapp T, Oláh L, Hölker I, Besselmann M, Tiesler C, Maeda K, Hossmann KA. GTPase RhoB: an early predictor of neuronal death after transient focal ischemia in mice. Mol Cell Neurosci. 2001 May;17(5):883-94.

97. Tury A, Mairet-Coello G, Poncet F, Jacquemard C, Risold PY, Fellmann D, Griffond B. QSOX sulfhydryl oxidase in rat adenohypophysis: localization and regulation by estrogens. J Endocrinol. 2004 Nov;183(2):353-63.

98. Uchida M, Enomoto A, Fukuda T, Kurokawa K, Maeda K, Kodama Y, Asai N, Hasegawa T, Shimono Y, Jijiwa M, Ichihara M, Murakumo Y, Takahashi M. Dok-4 regulates GDNF-dependent neurite outgrowth through downstream activation of Rap1 and mitogen-activated protein kinase. J Cell Sci. 2006 Aug 1;119(Pt 15):3067-77.

99. Ueki N, Kondo M, Seki N, Yano K, Oda T, Masuho Y, Muramatsu M. NOLP: identification of a novel human nucleolar protein and determination of sequence requirements for its nucleolar localization. Biochem Biophys Res Commun. 1998 Nov 9;252(1):97-102.

100. Umenishi F, Schrier RW. Induction of human aquaporin-1 gene by retinoic acid in human erythroleukemia HEL cells. Biochem Biophys Res Commun. 2002 May 10;293(3):913-7.

101. von Boxberg Y, Salim C, Soares S, Baloui H, Alterio J, Ravaille-Veron M, Nothias F. Spinal cord injury-induced up-regulation of AHNAK, expressed in cells delineating cystic cavities, and associated with neoangiogenesis. Eur J Neurosci. 2006 Aug;24(4):1031-41.

102. Walisko O, Schorn A, Rolfs F, Devaraj A, Miskey C, Izsvák Z, Ivics Z. Transcriptional activities of the Sleeping Beauty transposon and shielding its genetic cargo with insulators. Mol Ther. 2008 Feb;16(2):359-69.

103. Walther RF, Atlas E, Carrigan A, Rouleau Y, Edgecombe A, Visentin L, Lamprecht C, Addicks GC, Haché RJ, Lefebvre YA. A serine/threonine-rich motif is one of three nuclear localization signals that determine unidirectional transport of the mineralocorticoid receptor to the nucleus. J Biol Chem. 2005 Apr 29;280(17):17549-61.

104. Wang C, Kelly J, Bowen-Pope DF, Stiles CD. Retinoic acid promotes transcription of the platelet-derived growth factor alpha-receptor gene. Mol Cell Biol. 1990 Dec;10(12):6781-4.

105. Wang L, Watson DG, Lenox RH. Myristoylation alters retinoic acid-induced down-regulation of MARCKS in immortalized hippocampal cells. Biochem Biophys Res Commun. 2000 Sep 16;276(1):183-8.

106. Westphal NJ, Seasholtz AF. CRH-BP: the regulation and function of a phylogenetically conserved binding protein. Front Biosci. 2006 May 1;11:1878-91. Review.

107. Willard MD, Willard FS, Li X, Cappell SD, Snider WD, Siderovski DP. Selective role for RGS12 as a Ras/Raf/MEK scaffold in nerve growth factor-mediated differentiation. EMBO J. 2007 Apr 18;26(8):2029-40.

108. Xu LL, Shanmugam N, Segawa T, Sesterhenn IA, McLeod DG, Moul JW, Srivastava S. A novel androgen-regulated gene, PMEPA1, located on chromosome 20q13 exhibits high level expression in prostate. Genomics. 2000 Jun 15;66(3):257-63.

109. Yamada HY, Gorbsky GJ. Tumor suppressor candidate TSSC5 is regulated by UbcH6 and a novel ubiquitin ligase RING105. Oncogene. 2006 Mar 2;25(9):1330-9.

110. Yan F, Qian M, Yang F, Cai F, Yuan Z, Lai S, Zhao X, Gou L, Hu Z, Deng H. A novel pro-apoptosis protein PNAS-4 from Xenopus laevis: cloning, expression, purification, and polyclonal antibody production. Biochemistry (Mosc). 2007 Jun;72(6):664-71.

111. Yang H, Mattingly RR. The Ras-GRF1 exchange factor coordinates activation of H-Ras and Rac1 to control neuronal morphology. Mol Biol Cell. 2006 May;17(5):2177-89.

112. Yin Z, Haynie J, Williams BR, Yang YC. C114 is a novel IL-11-inducible nuclear double-stranded RNA-binding protein that inhibits protein kinase R. J Biol Chem. 2003 Jun 20;278(25):22838-45.

113. Yoon M, Spear PG. Disruption of adherens junctions liberates nectin-1 to serve as receptor for herpes simplex virus and pseudorabies virus entry. J Virol. 2002 Jul;76(14):7203-8.

114. Yoshida K, Yamada M, Nishio C, Konishi A, Hatanaka H. SNRK, a member of the SNF1 family, is related to low K(+)-induced apoptosis of cultured rat cerebellar granule neurons. Brain Res. 2000 Aug 11;873(2):274-82.

115. Yu G, Zerucha T, Ekker M, Rubenstein JL. Evidence that GRIP, a PDZ-domain protein which is expressed in the embryonic forebrain, co-activates transcription with DLX homeodomain proteins. Brain Res Dev Brain Res. 2001 Oct 24;130(2):217-30

116. Zheng C, Heintz N, Hatten ME. CNS gene encoding astrotactin, which supports neuronal migration along glial fibers. Science. 1996 Apr 19;272(5260):417-9.

117. Zhou H, Kim SA, Kirk EA, Tippens AL, Sun H, Haeseleer F, Lee A. Ca2+-binding protein-1 facilitates and forms a postsynaptic complex with Cav1.2 (L-type) Ca2+ channels. J Neurosci. 2004 May 12;24(19):4698-708.
