# Supplementary material for: Birdsong “Transcriptomics”: Neurochemical Specializations of the Oscine Song System
Source: PLoS One. 2008 Oct 20;3(10):e3440. doi: 10.1371/journal.pone.0003440 (PMC2563692; doi:10.1371/journal.pone.0003440)
Supplement: Table S1 — Primary FDR<0.05 HVC Markers. (0.08 MB PDF) [file pone.0003440.s002.pdf]

Table S1. Primary FDR < 0.05 HVC Marker.

Entrez Gene Nam	Gene Annotation	Genbank Accession No.	Gene Regulation	Literature* Cited
ABCC5	ATP-binding cassette sub-family C member 5	DV960769	+	9, 30
ADAM23	A disintegrin and metallopeptidase domain 23	CK305463, CK306577, DV961129	+	
ADSSL1	Adenylosuccinate synthetase 1	CK312450	+	
AHNAK2	Neuroblast differentiation-associated protein AHNAK	CK305980	+	24, 101
AIFM1	Apoptosis-inducing factor, mitochondrion-associated, 1	DV954850	+	
AIM1	Absent in melanoma 1, predicted	DV953279	+	
ALDH1A2	Retinal dehydrogenase 2 (RalDH2)	CK234972, DV957057	+	
ANK1	Ankyrin 1 isoform 3	DV958864	+	90
ANXA6	Annexin A6 (Annexin VI) Splice variant of DV958616	DV958615	+	6
ANXA6	Annexin A6 (Annexin VI) Splice variant of DV958615	DV958216	+	
ATP1B4	Sodium/potassium-transporting ATPase beta-4 chain	CK313848	+	
AYTL1	Acyltransferase like 1	CK306691	+	
C1orf34	Hypothetical protein DEME-6, KIAA0452	DV955796	+	50
C20orf3	Hypothetical protein LOC416715	DV950007	+	
C6orf168	Hypothetical protein LOC84553	DV953368	+	
CABP1	Calcium-binding protein 1 (CaBP1)	DV951925, DV959391	+	54, 117
CADPS2	Ca2+-dependent secretion activator isoform 3	DV955943	+	93
CALCR	Calcitonin receptor isoform b precursor	DV948514	+	
CAMK1D	Calcium/calmodulin-dependent protein kinase 1D	CK310981, CK316602	+	
CAMTA1	Calmodulin-binding transcription activator 1	CK313520	+	34
CD59	CD59 glycoprotein precursor (Membrane at	CK310319	+	
CD99L2	CD99 molecule-like 2	DV959159	+	92
CHGB	Chromogranin B (secretogranin 1) (CHGB)	CK308123	+	
CHRNA5	Cholinergic receptor nicotinic alpha 5	DV949835	+	
CHRNA7	Neuronal acetylcholine receptor protein, alpha-7	CK304602, DV957289	+	
COL21A1	Collagen type XXI alpha 1 precursor	CK304548	+	
COL4A2	Alpha 2 type IV collagen preproprotein	DV950044	+	
CPM	Carboxypeptidase M precursor	DV952396	+	
CRHBP	Corticotropin-releasing factor binding protein	DV945761, DV955207	+	106

CRISPLD1	Cysteine-rich secretory protein LCCL domain containing 1	DV952515	+	
CXCL14	Chemokine (C-X-C motif) ligand 14	DV959392	+	
DCN	Decorin precursor (Bone proteoglycan II)	CK304538, DV960642	+	
DIP2A	Similar to Disco-interacting protein 2 homolog A	CK316189	+	115
DNAPTP6	DNA polymerase-transactivated protein 6	DV951746	+	
DOK4	Docking protein 4	DV948997	+	98
DPP10	Inactive dipeptidyl peptidase 10	CK308156, CK312699	+	37
DPP6	Dipeptidyl-peptidase 6 isoform 1	DV956959	+	70
ENDOGL1	Endonuclease G-like 1 (Endo G-like)	DV958598	+	
FAM13A1	Family with sequence similarity 13 member A1	DV950254	+	
FASN	Fatty acid synthase	DV946694	+	
FNTM2	Fibronectin type III and transmembrane	CK234984, DV954885	+	3
FOSL2	Fos-like antigen 2	CK235813	+	
FST	Follistatin precursor	CK304072, CK309792	+	10
GABRE	Gamma-aminobutyric acid (GABA) A receptor, epsilon	DV945174	+	
GBAS	Protein NipSnap2	CK309317	+	
GPC3	Glypican 3	CK309542	+	64
GPI	Glucose-6-phosphate isomerase	DV961633	+	
HTR1F	5-hydroxytryptamine (serotonin) receptor 1F	CK301212, CK302146	+	
IL4R	Interleukin-4 receptor alpha-chain	DV954148	+	
INF2	Hypothetical protein LOC64423 isoform 1	DV953705	+	
ITGAX	ITGAX integrin, alpha X (comp 3 receptor 4 subunit)	DV953883	+	
KCNA1	Potassium voltage-gated channel subfamily A	CK234562	+	
KIAA1604	MKIAA1604 protein	DV949108	+	
LGMN	Legumain preproprotein	CK313657	+	
LMO2	LIM domain only 2 (rhombotin-like 1)	DV948088	+	
LMO3	LIM domain only 3	DV945566	+	
LOC400120	Hypothetical protein LOC418900	DV958430	+	
MAP1B	Microtubule-associated protein 1B isoform 2	DV961164	+	
MAP4	Microtubule-associated protein 4	DV960946	+	
MAPK11	Mitogen-activated protein kinase 11	CK310452	+	
MCF2	Mcf2 transforming sequence-like	DV952149	+	31, 46
MGC42105	Hypothetical protein (protein amino acid phosphorylation)	CK306856	+	

MUSTN1	Musculoskeletal, embryonic nuclear protein 1	DV958286	+	
NBL1	Neuroblastoma, suppression of tumorigenicity 1	DV950926	+	5
NDRG4	NDRG family member 4	CK311117	+	5, 33, 75
NEFH	Neurofilament heavy polypeptide 200kDa	CK235399	+	
NEFL	Neurofilament triplet L protein	CK304961	+	
NEFM	Neurofilament 3 medium	CK313443	+	
NETO1	Neuropilin and tolloid-like protein 1	DV957928	+	
NPY2R	Neuropeptide Y receptor type 2	CK315377	+	
NR3C2	Nuclear receptor subfamily 3 group C member 2	DV949530	+	57, 103
NRP1	Neuropilin 1 isoform a	DV955873	+	
NTS	Neurotensin/neuromedin N precursor	CK302282	+	
PARD3	Partitioning-defective protein 3 homolog	DV955889	+	
<u>PDE8B</u>	Phosphodiesterase 8B	DV960566	+	
PDZRN3	PDZ domain containing RING finger 3	CK304327	+	27, 44, 62
PGM1	Phosphoglucomutase-1	DV959732	+	
PLXNA4	Plexin-A4	DV951095	+	94
PPP4R2	Protein phosphatase 4 regulatory subunit 2	DV946429	+	
<u>PRDM11</u>	PR domain containing 11	DV959665	+	
PRKRIP1	PRKR interacting protein 1 (IL11 inducible)	DV946957	+	112
<u>PTGER4</u>	Prostaglandin E receptor 4 subtype	DV958814	+	
PTPRF	Protein tyrosine phosphatase receptor type F	DV959888	+	86
PVALB	Parvalbumin, muscle.	CK305573	+	
PYGB	Brain glycogen phosphorylase	CK313966	+	
QSOX6	Quiescin/sulfhydryl oxidase	DV957118	+	97
<u>RAB39</u>	Rab-related GTP-binding protein	DV957379	+	
RASGRP1	Ras guanyl releasing protein 1	CK316065	+	
RCAN2	Regulator of calcineurin 2	CK313417	+	25
RGS4	Regulator of G-protein signalling 4	CK314393	+	26, 83
RHOB	Rho-related GTP-binding protein RhoB	CK308435	+	63,67, 73, 96
RP6-213H19	Serine/threonine protein kinase MASK; STE20-like kinase	CK235627	+	5, 18, 66
S100B	S-100 calcium-binding protein beta subunit	DV950377	+	
SACS	Sacsin	CK303077	+	
SAP30L	Sin3A associated protein p30-like	CK307786	+	47, 60

SCUBE1	signal peptide-CUB domian-EGF-related 1	DV947442	+	
SCUBE1/2	Signal peptide CUB domain EGF-like 1	CK305644	+	32
SETD1B	SET domain containing 1B	CK315752	+	
SLC38A10	Hypothetical protein LOC124565 isoform a	DV946074	+	
SLC7A2	Solute carrier family 7 member 2 isoform 2	CK309001	+	
SLC8A1	Solute carrier family 8 (sodium/calcium)	DV947699	+	
SLC9A7	Sodium/hydrogen exchanger 7 (Na(+)/H(+)	DV950073, DV956252	+	
SNCG	Gamma-synuclein (Persyn)	DV945245, DV945607	+	39
SOX6	SRY (sex determining region Y)-box 5	CK301414	+	
ST8SIA4	ST8 alpha-N-acetyl-neuraminide A2,8-sialyltransferase 4	DV957771	+	4
STARD13	START domain containing 13 isoform alpha	DV961730	+	
STC2	stanniocalcin 2	CK235424	+	22
STK11IP	Serine/threonine kinase 11 interacting protein	DV960621	+	
STMN3	Stathmin-like 3	DV949164	+	41
SV2B	Synaptic vesicle glycoprotein 2b	CK302240	+	
SYCP1	Synaptonemal complex protein 1	CK305087, DV958591	+	
SYF2	SYF2 homolog RNA splicing factor isoform 1	CK306847	+	109
SYNC1	Syncoilin intermediate filament 1	DV953434	+	
SYNJ1	Synaptojanin 1 isoform a	CK313607	+	
TBR1	T-box brain gene 1	CK301650	+	20
THBS4	Thrombospondin-4 precursor	DV951570	+	
THOC4	THO complex 4	CK301480	+	
TMEM16E	Transmembrane protein 16E	DV947412	+	
TMEM74	Hypothetical proteins LOC239408, LOC157753	DV952526, DV956586	+	
TMTC1	ARG99 protein	CK315830	+	
TNR	Tenascin precursor	CK314049	+	12, 58, 69,84
TOMM70A	Translocase of outer mitochondrial membrane 70	DV949336	+	
TRIM8	Tripartite motif-containing 8	CK305942	+	
TSPAN9	Tetraspanin 9	DV945109	+	
UBE2E1	Ubiquitin-conjugating enzyme E2 E1 isoform 1	DV948876	+	
UTS2B	Prepro-urotensin II-related peptide	DV945629	+	
VLLH2748	Hypothetical protein VLLH2748	DV959083	+	
WASL	Wiskott-Aldrich syndrome gene-like protein	DV955503	+	

ZEB2	Zinc finger homeobox protein 1b	CK308008, DV946921	+	77, 78
ZNF533	Zinc finger protein 533	CK316105	+	
Unknown		CK302356	+	
Unknown		CK306005	+	
Unknown		none	+	
Unknown		DV945704	+	
Unknown		DV960681	+	
Unknown		CK234517	+	
Unknown		DV957618	+	
Unknown		DV951785	+	
Unknown		CK311256	+	
Unknown		CK312976	+	
Unknown		DV951875	+	
Unknown		DV961502	+	
Unknown		DV956767	+	
Unknown		CK312284	+	
Unknown		CK304898	+	
Unknown		DV958743	+	
Unknown		DV950322	+	
Unknown		CK311619	+	
Unknown		DV945166	+	
Unknown		DV959261	+	
Unknown		DV960469	+	
Unknown		CK306963	+	
Unknown		CK307876	+	
Unknown		DV950296	+	
Unknown	May be an RT.	DV949717	+	
Unknown		DV952548	+	
Unknown		CK306373	+	
Unknown		CK315619	+	
Unknown		DV948470, DV947307	+	
Unknown		DV958960	+	
ABCA1	ATP-binding cassette sub-family A (ABC1)	DV946486	-	

ADAMTS8	ADAM metallopeptidase with thrombospondin type 1	CK304156	-	
ANK3	Ankyrin 3 epithelial isoform 1	DV959675	-	
ANKRD12	Ankyrin repeat domain protein 12 (GAC-1 protein)	DV955183	-	
ANTXR2	Anthrax toxin receptor 2	DV958955	-	
AQP1	Aquaporin-1, complete	DV948000	-	100
ARHGDIB	Rho GDP-dissociation inhibitor 2	CK305571	-	52, 87
ARL5B	ADP-ribosylation factor-like 5B	CK305762	-	
ARPP21	Cyclic AMP-regulated phosphoprotein 21 kD	CK308798	-	
ASTN1	Astrotactin isoform 1	DV960317	-	116
ATP8A1	ATPase aminophospholipid transporter	CK304944, CK311916	-	
B3GNT1	UDP-GlcNAc:BGal B-1,3-N-acetylglucosaminyltransferase 1	CK312440	-	
BAI3	Brain-specific angiogenesis inhibitor 3	CK313813	-	
BMPR2	Bone morphogenetic protein receptor type II	CK316243	-	55
C14orf147	Chromosome 14 open reading frame 147	DV955829	-	
C1orf187	Hypothetical protein UNQ3119; AGPA3119; MGC117222	CK235460	-	
C1QL3	Complement component 1 q subcomponent-like 3	DV960345	-	
C3orf39	Glycosyltransferase	DV948284, CK302348, CK312228	-	
C5orf30	Hypothetical protein LOC90355	DV956689	-	
CACNA1G	Calcium channel voltage-dependent T type	CK306076	-	80
CACNG4	Voltage-dependent calcium channel gamma-subunit	DV953758, DV947575	-	
CAMK2D	Calcium/calmodulin-dependent protein kinase II delta	CK234141	-	5
CAP2	CAP adenylate cyclase-associated protein 2	CK304498	-	
CCK	Cholecystokinins precursor (CCK) [Contai	CK302967	-	
CHODL	Chondrolectin precursor	CK308108, CK304078	-	
CHST11	Carbohydrate sulfotransferase 11	CK317232	-	43
CHST2	Carbohydrate (N-acetylglucosamine-6-O)	CK308681	-	
CLMN	Calponin like transmembrane domain protein	CK310744	-	36, 69, 95
CNOT6	CCR4-NOT transcription complex subunit 6	DV946818	-	
CPNE2	Copine-2 (Copine II)	DV960053, DV960587	-	
CPNE8	Copine-8 (Copine VIII)	CK235787	-	
CREB1	cAmp response element binding protein 1	DV953443	-	
CYP19A1	Aromatase; P450, family 19, subfamily A	DV951351, DV954113	-	
CYP1B1	Cytochrome P450 family 1 subfamily B	DV958401	-	

DACH2	Dach2 protein	DV959755	-	
DACT1	Dapper 1 isoform 2	CK311605	-	
DHRS2	Dehydrogenase/reductase SDR family member 2	CK313067	-	5, 23
DOCK4	Dedicator of cytokinesis 4	DV953606	-	29
EML5	echinoderm microtubule associated protein like 5	CK317261	-	
ENOX1	ecto-NOX disulfide-thiol exchanger 1	DV961675	-	
EPB41L2	Erythrocyte membrane protein band 4.1-like 2	DV961341	-	16
FAM18A	Hypothetical protein LOC780776	CK312908	-	
FAM152A	FAM152A	CK235192	-	110
FAM5C	BMP and Retinoic Acid Inducible Protein-3	DV954478	-	91
FAM84A	NSE1	DV951531	-	45
FJX1	Four-jointed protein (fj gene).	DV952932	-	
FNBP1	Formin binding protein 1	CK305640	-	
FSTL4	Follistatin-like 4	CK312465	-	
GALNTL	UDP-N-acetyl-alpha-D-galactosamine:polypeptide	DV961240	-	
GAS7	Growth arrest-specific 7 isoform c	DV953799	-	15, 19, 61
GLRA2	Glycine receptor alpha 2	CK307985, DV947472, DV956508, D	-	
GPR173	Probable G-protein coupled receptor 173	CK311142	-	
GPR177	G protein-coupled receptor 177	CK304903	-	
GPR98	G protein-coupled receptor 98 precursor	CK306286	-	
GRIA4	Glutamate receptor ionotrophic AMPA 4	DV961576	-	
GRIK2	Glutamate receptor 6 isoform 1 precursor	DV956587	-	
GRM1	Glutamate receptor, metabotropic 1	DV960061	-	
GRM5	Glutamate receptor, metabotropic 5	CK301242	-	
GRRP1	GRRP1	CK309164	-	
HMG2L1	High mobility group box 2-like 1	CK302461	-	102
HOPX	Homeodomain-only protein (Odd homeobox protein 1)	DV951479	-	
HPCAL1	Hippocalcin-like 1	CK316984	-	
HRH3	Histamine H3 receptor	DV959303	-	
HS3ST1	Heparan sulfate glucosamine 3-O-sulfotra	CK304125, DV959802*	-	
KCNC2	Potassium voltage gated channel Shaw-related	CK311592	-	
KCND2	Potassium voltage-gated channel Shal-related	DV956417	-	
KCNF1	Potassium voltage-gated channel subfamily F	CK306387, DV956078	-	

KCNIP1	Kv channel-interacting protein 1	CK305253, DV951220*	-	
KCNK10	Potassium channel subfamily K member 10	DV957671	-	
KCNK5	Potassium channel subfamily K member 5	DV953320	-	
KIAA1549	Hypothetical protein LOC512679	DV948066, DV946365	-	
KLHL2	Kelch-like protein 2 (Actin-binding protein Mayven)	CK311719	-	13, 38
LIN7A	Lin-7 homolog A	CK307101	-	11
LOC345222	Hypothetical protein LOC345222	CK310874	-	
LOC418413	Hypothetical protein LOC418413	DV960503	-	
LOC771589	Hypothetical protein LOC771589	DV946404	-	
LOC771914	Hypothetical protein LOC771914	DV947077	-	
LPPR4	Lipid phosphate phosphatase family	DV955659	-	
LRRIQ1	Leucine-rich repeats and IQ motif containing 1	DV956844	-	
MAP7	Microtubule-associated protein 7	DV960895	-	
MARCKS	Myristoylated alanine-rich C-kinase substrate	CK312689, DV955758	-	14, 35, 56, 105
MEGF6	EGF-like-domain multiple 3	CK315335	-	
MEIS1	Meis1 homolog	CK311803	-	21, 79
MMEL1	Membrane metallo-endopeptidase	DV950386	-	
MMD2	Monocyte to macrophage	CK311668	-	
MPPED1	Metallophosphoesterase domain containing 1	DV946651	-	
MTPN	Myotrophin	CK304305	-	
MYCN	N-myc proto-oncogene protein	CK308936	-	40
MYO1B	Myosin Ib	CK235788, CK305346, DV952542	-	
NAP1L4	Nucleosome assembly protein 1-like 4	CK315498	-	
NEUROD1	Neurogenic differentiation 1	DV945348	-	
NOL4	Nucleolar protein 4	CK234421, CK306792	-	99
NOP5/NOP5	Nucleolar protein NOP5/ NOP58	DV957014	-	65, 71
NRXN3	Neurexin 3 isoform alpha precursor	CK317422, DV957019	-	
NSUN6	NOL1/NOP2/Sun domain family, member 6	CK314629	-	
Odz3	Odd Oz/ten-m homolog 3	DV947941	-	
PAIP1	Poly(A) binding protein interacting protein 1	DV953504	-	
PCBP3	Poly(rC) binding protein 3	DV951913	-	
PDGFRA	Platelet-derived growth factor receptor alpha	DV949723	-	2, 105
PHLDB2	Pleckstrin homology-like domain family B	CK301530	-	53

PLCXD3	Phosphatidylinositol-specific phospholipase C	DV948267	-	
PLD1	Phospholipase D1 phophatidylcholine-specific	CK305385	-	42, 74
PLXNA1	Plexin A1	DV960964	-	
PPFIBP1	PTPRF interacting protein binding protein 1	DV958897	-	89
PRKAR2B	cAMP-dependent protein kinase regulatory	CK313635, CK313713	-	
PRKCD	Protein kinase C delta	CK316407	-	51, 76, 81
PRR5	Proline rich 5 (renal)	DV945125	-	
PTER	Phosphotriesterase related	CK302959, CK305472	-	
PTPRZ1	Phosphotyrosyl phosphatase	CK314246, CK315330	-	
PVRL1	Poliovirus receptor related protein 1	CK304335	-	48, 113
RAB36	Ras-related protein Rab-36	CK303949	-	
RASD2	RasD family member 2	CK301759, CK307974	-	
RASGRF1	Ras protein-specific guanine	CK305981	-	7, 111
RASL11A	Ras-like, family 11, member A	CK316558	-	
RASL12	Ras-like, family 12	CK304375, CK315695	-	
RELN	Reelin isoform a	CK316482	-	72
RFTN1	Raft-linking protein	DV951317	-	
RGS12	Regulator of G-protein signalling 12 isoform 2	CK308262	-	82, 85, 107
RGS16	Regulator of G-protein signalling 16	DV952832	-	
RPL18A	Ribosomal protein L18a	DV949155	-	
RPRML	Reprimo-like	DV949746	-	
RSPO3	R-spondin 3	DV947295	-	
SCN3B	Sodium channel, voltage-gated, type III, beta	CK301607, CK315207	-	
SEMA3A	Semaphorin 3A	CK304131	-	
SEMA6A	Semaphorin 6A domain transmembrane domain	CK308635	-	
SHC1	SHC transforming protein 1	DV961745	-	68
SHC3	Src homology 2 domain containing transforming protein	DV954662	-	17
SLC32A1	Solute carrier family 32 member 1	CK306580	-	
SNRK	SNF Related Kinase	CK235150	-	8, 88, 114
SPSB4	PRY domain-containing SOCS box protein	CK305838	-	
SYT10	Synaptotagmin X	DV959162	-	
TAC1	Tachykinin precursor 1	CK301285, DV958953	-	
TCOF1	Treacher Collins-Franceschetti syndrome 1	DV961952	-	

TIMM23	Translocase of inner mitochondrial membrane 23	DV954624	-	
TIMP3	Metalloproteinase inhibitor 3 precursor	DV945697, CK317419	-	
TLL1	Tolloid-like 1	CK306427	-	
TMEM158	Ras-induced senescence 1	DV959600	-	
TMEPAI	Transmembrane prostate androgen-induced protein	DV946041	-	108
TNFAIP8L3	Tumor necrosis factor alpha-induced protein	DV945072, DV956728	-	
TOX2	TOX high mobility group box family member 2	DV955585	-	
TRIB2	Tribbles homolog 2	CK301526, CK301680	-	59
TRP53i11	Trp53 inducible protein 11	CK312269	-	
TUBGCP5	Gamma-tubulin complex component 5	CK306882	-	
UBE2D3	Ubiquitin-conjugating enzyme E2 D3	CK313794	-	28
UNC5C	Netrin receptor	DV952298	-	
USP46	Ubiquitin specific peptidase 46	DV952053	-	
ZDHHC22	Zinc finger DHHC domain containing 22	CK308396, DV956166	-	
ZNF423	Zinc finger protein 423	DV958428	-	49
Unknown	Contaminent complex 1 (zebra finch)	none	-	
Unknown		CK235923	-	
Unknown		CK311781	-	
Unknown		CK301827	-	
Unknown		CK302798	-	
Unknown		CK316508	-	
Unknown		CK315179	-	
Unknown		CK306924	-	
Unknown		CK307201	-	
Unknown		CK308337	-	
Unknown	RIKEN cDNA C630035N08, contaminent complex 2	CK309395	-	
Unknown		DV945794	-	
Unknown		DV946239	-	
Unknown		DV946874	-	
Unknown		DV949079	-	
Unknown		DV950695	-	
Unknown		DV951401	-	
Unknown		DV952261	-	

Unknown		DV953500	-	
Unknown		DV953909	-	
Unknown		DV954593	-	
Unknown		DV954727	-	
Unknown		DV955038	-	
Unknown		DV955453	-	
Unknown		DV955986	-	
Unknown		DV959089, DV954221	-	
Unknown		DV960158	-	
Unknown		DV960513	-	
Unknown		DV960601	-	
Unknown		DV960989	-	
Unknown		DV961039	-	

Tentative gene identifications are underlined (see methods for details).

* See References S1 for references cited in this table.
