# Supplementary material for: Birdsong “Transcriptomics”: Neurochemical Specializations of the Oscine Song System
Source: PLoS One. 2008 Oct 20;3(10):e3440. doi: 10.1371/journal.pone.0003440 (PMC2563692; doi:10.1371/journal.pone.0003440)
Supplement: Table S2 — Secondary List of Differential Expressed HVC Markers. (0.06 MB PDF) [file pone.0003440.s003.pdf]

Table S2. Secondary List of Differential Expressed HVC Markers

Entrez Gene Name	Annotated Gene Name	Gene Regulation	Genbank Accession
CELL ADHESION AND CYTOSKELETAL ORGANIZATION			
CDH1	Cadherin 1, type 1, E-cadherin (epithelial)	+	DV947236
COL2A1	Procollagen, type II, alpha 1	+	CK304054
COL6A1	Collagen, type VI, alpha 1	+	CK303649
COL10A1	Collagen, type X, alpha 1	+	CK308288
COL12A1	Collagen, type XII, alpha 1	+	CK312236
CTNNA1	Catenin (cadherin-associated protein), alpha 1, 102kDa	+	DV955801
SSX2IP	Synovial sarcoma, X breakpoint 2 interacting protein	-	DV949510
AXON GUIDANCE AND NEURITE OUTGROWTH			
PLXNB1	Plexin B1	-	CK314407
NEUROTRANSMISSION AND CELLULAR EXCITABILITY			
CACNA1D	Calcium channel, voltage-dependent, L type, alpha 1D subunit	-	DV952804
CHRM4	Cholinergic receptor, muscarinic 4	+	DV955103
KCNAB1	Potassium voltage-gated channel, shaker-related subfamily, beta 1	+	CK303535
KCNC1	Potassium voltage-gated channel, Shaw-related subfamily, member 1	+	DV948250
KCNC3	Potassium voltage gated channel, Shaw-related subfamily, member 3	+	DV953393
KCNG1	Potassium voltage-gated channel, subfamily G, member 1	-	CK315176
KCNS2	Potassium voltage-gated channel, delayed-rectifier, subfamily S2	-	CK315983
KCNV1	Potassium channel, subfamily V, member 1	+	DV947438
CELL PROLIFERATION, SURVIVAL, AND DEATH			
ACVR1	Activin A receptor, type I	-	DV961653
GADD45G	Growth arrest and DNA-damage-inducible, gamma	-	CK304105
LTBP1	Latent transforming growth factor beta binding protein 1	+	CK315502
TRAIP	TRAF interacting protein	-	DV960928
TGFBR2	TGF-beta receptor type II	+	DV956964
RETINOIC ACID SIGNALING			

CBX3	Chromobox homolog 3	-	CK303377
CXCR4	Chemokine (C-X-C motif) receptor 4	+	CK303587
DBC1	Deleted in bladder cancer 1	+	CK307950
FRZB	Frizzled-related protein	+	DV960300
GTF2H1	General transcription factor IIH, polypeptide 1	+	DV955862
HSD17B11/13	Hydroxysteroid (17-beta) dehydrogenase 11/13	-	DV951599
JAG1	Jagged 1 (Alagille syndrome)	-	CK308079
JUN	jun oncogene	-	DV945400
NDRG1	N-myc downstream regulated gene 1	+	CK316108
RAI1	Retinoic acid-induced protein 1	+	CK315235
RAI14	Retinoic acid induced 14	-	DV961572
SFRP2	Secreted frizzled-related protein 2	+	DV959544
