# Supplementary material for: Birdsong “Transcriptomics”: Neurochemical Specializations of the Oscine Song System
Source: PLoS One. 2008 Oct 20;3(10):e3440. doi: 10.1371/journal.pone.0003440 (PMC2563692; doi:10.1371/journal.pone.0003440)
Supplement: Table S3 — Previously Confirmed Markers of HVC. (0.04 MB PDF) [file pone.0003440.s004.pdf]

Table S3. Previously Confirmed Markers of HVC.

Entrez Gene Name	Annotated Gene Name	Genbank Accession	Evidence of Enrichment
ALDH1A2	Retinal dehydrogenase 2 (zRalDH)	DV957057, CK234972	Denisenko-Nehrbass et al., 2000
CAMTA1	Calmodulin-binding transcription activator 1	CK313520	Li et al., 2007
NEFL	Neurofilament triplet light (NF-M)	CK304961	Velho et al., 2006
NEFM	Neurofilament triplet medium (NF-L)	CK313443	Velho et al., 2006
FNTM2	Phantom2	DV954885, CK234984	Agate et al., 2007
IGF2*	Insulin growth factor 2	CK308292	Holzenberger et al., 1997
KCNA1*	Voltage-gated potassium channel C3	DV953393	Velho and Mello, unpublished
SNCA*	Alpha-synuclein	DV950418	George et al., 1995
GRM8*	Glutamate receptor, metabotroic 8	CK314839	Li et al., 2007
PVALB	Parvalbumin	CK305573	Wild et al., 2005
CYP19A1	P450 aromatase	DV954113, DV951351	Chen et al., 1995
GRIA2*	Glutamate receptor, ionotropic AMPA 2	CK234665, CK306281, CK312715	Wada et al., 2004
GRIA3*	Glutamate receptor, ionotropic AMPA 3	DV945013	Wada et al., 2004
GRIA4*	Glutamate receptor, ionotropic AMPA 4	CK316778	Wada et al., 2004
GRIK2	Glutamate receptor, ionotropic kainate 4	DV956587	Wada et al., 2004
GRM1	Glutamate receptor, metabotroic 1	DV960061	Wada et al., 2004
GRM5	Glutamate receptor, metabotroic 5	CK301242	Wada et al., 2004
CHN1*	N-chimaerin	CK312097	Clayton and George, 1992
RELN	Reelin isoform	CK316482	Li et al., 2007
GRM3*	Glutamate receptor, metabotroic 3	CK301471, CK317241, CK308692, [illegible]	Wada et al., 2004
GRIK3*	Glutamate receptor, ionotropic kainate 3	CK234667, DV946752	Wada et al., 2004
GRIN1*	Glutamate receptor, ionotropic NMDA 1	CK311421	Wada et al., 2004
DBC1*	Deleted in bladder cancer 1	CK307950	Wood et al., 2008
HSD17B11/13*	Hydroxysteroid (17-beta) dehydrogenase 11/13	DV951599	Wood et al., 2008

Tentative gene identifications are underlined (see methods for details).

* Gene was present on the secondary gene list (Supplementary Table 2).
