# Supplementary material for: Birdsong “Transcriptomics”: Neurochemical Specializations of the Oscine Song System
Source: PLoS One. 2008 Oct 20;3(10):e3440. doi: 10.1371/journal.pone.0003440 (PMC2563692; doi:10.1371/journal.pone.0003440)
Supplement: Table S4 — Microarray validations by in situ hybridization (0.08 MB PDF) [file pone.0003440.s005.pdf]

Table S4. Microarray validations by *in situ* hybridization

Entrez Name	Genbank Accession	Annotated Gene Name	Array Predict.	Stat. Confirm.	Fold † Enrich.	Expression in Song Nuclei $^{\alpha}$			
						HVC	RA	LMAN	X
S100B	DV950377	S-100 calcium-binding protein b-subunit	+	P < 0.01	10.0	+	+	+	+
C14orf173	DV953705	Inverted Formin 2	+	P < 0.001	4.1	+	ND	+	ND
MUSTN1	DV958286	Musculoskeletal, embryonic nuclear 1	+	P < 0.01	2.7	+	ND	ND	ND
CADPS2	DV955943	Ca^{2+}-dependent secretion activator 3	+	P < 0.05	2.6	+	ND	ND	+
AIM1	DV953279	Absent in melanoma 1, predicted	+	P < 0.001	2.6	+	ND	ND	ND
ADSSL1	CK312450	Adenylosuccinate synthetase 1	+	P < 0.001	2.2	+	ND	+	ND
HTR1F	CK302146	Serotonin Receptor 1F	+	P < 0.05	2	+	ND	ND	ND
NRP1	DV955873	Neuropilin 1	+	√	1.9	+	ND	ND	+
MAP4	DV960946	Microtubule-associated protein 4	+	P < 0.05	1.8	+	+	+	-
Unknown	DV947307	Unknown	+	√	1.7	+	ND	ND	+
PTGER4	DV958814	Prostaglandin E receptor 4 subtype EP4	+	√	1.7	+	ND	ND	ND
CHRNA7	DV957289	Neuronal acetylcholine receptor a-7	+	P < 0.05	1.6	+	ND	ND	ND
MAPK11	CK310452	Mitogen-activated protein kinase 11	+	P = 0.08	1.6	+	ND	+	ND
CRHBP	DV945761	Corticotropin-releasing factor binding p	+	√	1.5	+	ND	ND	ND
SLC7A2	CK309001	Solute carrier family 7 member 2 iso 2	+	P < 0.01	1.4	+	ND	ND	ND
CHRM4*	DV955103	Muscarinic acetylcholine receptor M4	+	√	1.4	+	ND	ND	+
NTS	CK302282	Neurotensin/neuromedin N precursor	+	P < 0.05	1.3	+	ND	ND	+
CHRNA5	DV949835	Cholinergic receptor nicotinic alpha-5	+	√	1.3	+	ND	-	+
FST	CK304072	Follistatin	+	√	1.2	+	ND	ND	ND
Unknown	CK315619	Unknown	+	√	1.2	+	ND	ND	ND
PLXNA4	DV951095	Plexin-4A	+	√	1.2	+	ND	ND	ND
SEMA3A	CK304131	Semaphorin-3A	-	√	-2.1	-	-	ND	ND
RSP03	DV947295	R-spondin 3	-	P < 0.05	-2.2	-	+	-	ND
GLRA2	DV947472	Glycine receptor alpha 2	-	P < 0.01	-2.5	-	-	ND	ND
CHRNA2*	DV955395	Neuronal acetylcholine receptor, a-2	ND	√	ND	ND	ND	ND	ND
CHRNA2/4*	DV950553	Neuronal acetylcholine receptor, a-2/4	ND	√	ND	ND	ND	ND	ND
CHRM2*	DV954101	Muscarinic acetylcholine receptor M2	ND	√	ND	ND	ND	ND	ND
CRISPLD1	DV952515	Cysteine-rich secretory protein LCCL	+	*No Signal*	ND	*No Signal*			
BMPR2	CK316243	Bone Morphogenic Protein Receptor 2A	-	*No Signal*	ND	*No Signal*			

Tentative gene identifications are underlined (see methods for details).

† Average fold-differences in optical density (i.e. expression) over HVC versus Shelf measured from *in situ* autoradiograms (minimum n=2)

α Expression relative to surrounding tissue (i.e. HVC vs Shelf; RA vs A; LMAN vs N; X vs St); ND = No difference

β Abbreviations: H, Hyperpallium; M, Mesopallium; N, Nidopallium; A, Arcopallium; L2a, field L2a; Bas, Basorostralis, Nif, Nucleus interfacialis; V, ventricle; St, Striatum

* Visual confirmation, but sample size too small for ANOVA.

Brain Areas [β] Enriched
H, L2a, Bas, V
St (-)
M
Bas
H
M
M
Bas
St (+)
M
St (-)
M, H, St (-)
M
M
St (+)
No Signal
No Signal
