# Supplementary material for: Birdsong “Transcriptomics”: Neurochemical Specializations of the Oscine Song System
Source: PLoS One. 2008 Oct 20;3(10):e3440. doi: 10.1371/journal.pone.0003440 (PMC2563692; doi:10.1371/journal.pone.0003440)
Supplement: Table S5 — Extended search Genelist (ND). (0.06 MB PDF) [file pone.0003440.s006.pdf]

Table S5. Extended search Genelist (ND)

Entrez Name	Annotated Gene Name	Gene Regul.	Genbank Accession
CELL ADHESION AND CYTOSKELETAL ORGANIZATION			
COL3A1	Collagen, type III, alpha 1	ND	DV955812
COL5A1	Collagen, type V, alpha 1	ND	DV959671
COL7A1	Collagen, type VII, alpha 1	ND	CK311460
COL11A1	Collagen, type XI, alpha 1	ND	DV952180
COL13A1	Collagen, type XIII, alpha 1	ND	DV953159
CTNNA2	Catenin (cadherin-associated protein), alpha 2	ND	DV957691
CTNNB1	Catenin (cadherin-associated protein), beta 1	ND	CK310135, CK311500
DLG1	Discs large homolog 1 isoform 1	ND	DV949769
ITGB1	Integrin beta 1	ND	DV961664
ITGB3	Integrin beta 3	ND	DV955122
ITGB5	Integrin beta-5	ND	DV954283
ITGB8	Integrin beta 8	ND	DV961966
PDGFA	Platelet-derived growth factor alpha polypeptide	ND	DV959989, DV950799
PDGFC	Platelet derived growth factor C	ND	DV957068
RAC1	Ras-related C3 botulinum toxin substrate	ND	CK315716, CK314138
ROCK1	Rho-associated coiled-coil containing protein	ND	DV947676
LIMK1	LIM domain kinase 1	?	Not on array
AXON GUIDANCE AND NEURITE OUTGROWTH			
PLXNA2	Plexin A2	ND	CK311523
NEUROTRANSMISSION AND CELLULAR EXCITABILITY			
CHRNA2	Cholinergic receptor, nicotinic, alpha 2 (neuronal)	ND	DV950553
CHRNA4	Cholinergic receptor, nicotinic, alpha 4	ND	DV955395
CHRNA9	Cholinergic receptor, nicotinic, alpha 9	ND	CK303364
CHRM2	Cholinergic receptor, muscarinic 2	ND	DV954101
HTR1B	5-hydroxytryptamine (serotonin) receptor 1B	ND	CK303512
HTR7A	5-hydroxytryptamine (serotonin) receptor 7A	ND	CK310918
KCNA4	Potassium voltage-gated channel, shaker-related 4	ND	DV957069, DV952065
KCNA6/7	Potassium voltage-gated channel, shaker-related 6 or 7	ND	DV960691
KCNQ5	Potassium voltage-gated channel, KQT-like 2	ND	DV946128
CHRNA1	Cholinergic receptor, nicotinic, alpha 1	?	Not on array.
CHRNA3	Cholinergic receptor, nicotinic, alpha 3	?	Not on array.
CHRNA6	Cholinergic receptor, nicotinic, alpha 6	?	Not on array.
CHRNA8	Cholinergic receptor, nicotinic, alpha 8	?	Not on array.
CHRNB1	Cholinergic receptor, nicotinic, beta 1	?	Not on array.
CHRNB2	Cholinergic receptor, nicotinic, beta 2	?	Not on array.
CHRNB3	Cholinergic receptor, nicotinic, beta 3	?	Not on array.
CHRM1	Cholinergic receptor, muscarinic 1	?	Not on array.
CHRM3	Cholinergic receptor, muscarinic 3	?	Not on array.
CHRM5	Cholinergic receptor, muscarinic 5	?	Not on array.

CELL PROLIFERATION, SURVIVAL, AND DEATH			
ACVR2A	Activin A receptor, type IIA	ND	CK234683, DV956167
AIFM1	Apoptosis-inducing factor, mitochondrion-associated 1	ND	CK235030
BFAR	Bifunctional apoptosis regulator	ND	DV960865
CASP3	Caspase 3, apoptosis-related cysteine peptidase	ND	CK304919, CK310167
FAM130A2	TGF beta induced apotosis protein 2	ND	DV945583
GDF3	Growth differentiation factor 3	ND	CK310219
NOG	Noggin	ND	DV959546
PDCD2	Programmed cell death 2	ND	DV952222, CK311418
PDCD4	Programmed cell death 4	ND	CK313545, DV960500
PDCD5	Programmed cell death 5	ND	CK302153
PDCD6	Programmed cell death 6 interacting protein	ND	CK316110
PDGFA	Platelet-derived growth factor A chain p	ND	DV950799
SMAD1	SMAD family member 1	ND	CK317055
SMAD2	SMAD family member 2	ND	DV952451
SMAD6	SMAD family member 6	ND	CK303933
SMAD9	SMAD family member 9	ND	DV954742
TINP1	TGF beta-inducible nuclear protein 1	ND	CK316377
AMHR2A	Anti-Mullerian hormone receptor, type II	?	Not on array.
BMPR1A	Bone morphogenetic protein receptor, type 1	?	Not on array.
BMPR1B	Bone morphogenetic protein receptor, type 1	?	Not on array.
INHBA	Inhibin, beta A (a.k.a. Activin)	?	Not on array.
INHBB	Inhibin, beta B (a.k.a. Activin)	?	Not on array.
INHBC	Inhibin, beta C (a.k.a. Activin)	?	Not on array.
INHBE	Inhibin, beta E (a.k.a. Activin)	?	Not on array.
SMAD3	SMAD family member 3	?	Not on array.
SMAD4	SMAD family member 4	?	Not on array.
SMAD5	SMAD family member 5	?	Not on array.
SMAD7	SMAD family member 7	?	Not on array.
SMAD7	SMAD family member 7	?	Not on array.
TGFB3	Transforming growth factor, beta 3	?	Not on array.

Tentative gene identifications are underlined (see methods for details).
